# Perceived Organizational Democracy and Associated Factors: A Focused Systematic Review Based on Studies in Turkey

**DOI:** 10.3389/fpsyg.2022.767469

**Published:** 2022-04-15

**Authors:** Tahsin Geçkil

**Affiliations:** Department of Logistic Management, Faculty of Applied Science, Necmettin Erbakan University, Konya, Turkey

**Keywords:** organizational democracy, organizational democracy scale, business employee, participation in management, systematic review, demographic characteristics of employee, Turkey

## Abstract

This review study provides an opportunity to look at the level of organizational democracy (OD) that a large sample of private- and public-sector employees in an emerging market (Turkey) perceive. The focused systematic review includes empirical studies examining employees' level of OD and associated work and organizational psychological variables, using the Organizational Democracy Scale (ODS) in Turkey. This paper includes studies published between January 2014 and April 2021 in the Google Academic, Dergipark, and Ulakbim databases and on the Turkish National Thesis Center website. From a total of 1,778 records, 37 empirical studies meeting the inclusion criteria were included (with a total of *N* = 10,370 employees). Of these studies, 67.6% are published articles and manuscripts, 24% are unpublished dissertations, and 43.2% of the studies took place in the public sector. The results suggest that the level of employees' perceived OD was slightly above the medium level (mean: 3.30 ± 81), and the scores of the private-sector employees are higher than those of the public employees. Further, empirical associations between OD and 21 different outcome variables are reported and discussed. To varying extents, significant positive correlations were found between the level of employees' perceived OD and positive organizational variables, such as organizational citizenship behavior, organizational commitment, psychological capital, and job satisfaction. On the other hand, negative significant relationships occurred between OD and negatively evaluated organizational variables, such as job stress and organizational depression. The results of this study support the importance of organizational democracy as a management approach.

## Introduction

Organizational democracy (OD) refers to the participation of members of an organization in its management and processes (Harrison and Freeman, 2004). This participation is mandatory, continuous, broad-based, and institutionalized employee involvement, not temporary or occasional (Weber et al., 2020). Democracy in the workplace concerns sharing organizational decisions, greater employee autonomy, and strategic orientation (Drucker, 1999). Organizational Democracy promotes human development, increases a sense of political effectiveness, and reduces alienation (Kerr, 2004). The concept of OD serves to describe all types of management, from non-authoritarian to employee-managed or participatory firms (Cheney, 1995; Harrison and Freeman, 2004; Kerr, 2004; Weber et al., 2009; Yazdani, 2010; Unterrainer et al., 2011; Geckil and Tikici, 2016). OD produces individual outputs with the participation of employees in democratic decision-making processes. In addition, it creates the democratic organization by arranging its structure and processes, thus providing the desired organizational outputs. It also enables the employees of the enterprise to experience political activity, providing access to social outputs.

Harrison and Freeman (2004) define OD as “any action, structure, or process that increases the power of a broader group of people to influence the decisions and activities of an organization can be considered a move toward democracy” (p. 49). The process concept identifies how to embed democratic principles in the organizational structure—in other words, the democratization of organizations. A democratic organization is not an organization whose conditions are standard everywhere and in every situation. There may be democratic structures whose democratic principles occur on different levels. Adoption of participatory management practices at the organizational level, tolerance in the face of criticism, creation of a transparent, fair, and egalitarian structure, and the establishment of accountability as a rule express an advanced democratic organization. Requiring shared administrative power ensures the permanence of democratic principles.

Some of the researchers working on organizational democracy tend to explain it as participation in decisions and management. Others argue that organizational democracy relates to both the economic and social aspects of organizations and affects democratic tendencies and practices in social life. Moreover, organizational democracy is not only about organizational life but also about democratic attitudes and behaviors in social life.

In addition to its social, managerial, cultural, and environmental impacts on the organization (Pircher-Verdorfer et al., 2012), researchers also consider OD an important determinant of various expected organizational outcomes, including increased shareholder engagement and enhanced innovation, as well as improved organizational performance (Harrison and Freeman, 2004; Geçkil, 2017; Han and Garg, 2018). Democratic practices in organizational life eliminate unprofessional behaviors and increase labor efficiency (Yazdani, 2010). Moreover, they help to improve the morale of the workforce (Sagie and Koslowsky, 2000), provide better control over organizational structure and processes (Foley and Polanyi, 2006), and help to renew organizational structure and practices (Harrison and Freeman, 2004; Yazdani, 2010).

The term “organizational democracy” entered the management literature in 1897, via Sidney and Beatrice Webb (Müller-Jentsch, 2008). Although OD has appeared in the literature for more than a century, studies on its measurement have intensified over the last decade. One of the first examples of scale-development studies on the measurement of OD was by Weber and Unterrainer (2012), based on earlier studies by the IDE International Research Group (1981) and Heller et al. (1998). Later, the studies of OD scale development by Geçkil and Tikici (2015) and Ahmed et al. (2019) contributed to the literature. A generally accepted standard definition of the concept of OD is not found in the research literature, due to differences in individual, organizational, and social characteristics; thus, declaring a consensus in the literature on the dimensions of OD is difficult. Yazdani (2010) examines OD in two dimensions, Weber and Unterrainer (2012) in two, Geçkil and Tikici (2015) in seven, Vopalecky and Durda (2017) in 11, and Ahmed et al. (2019) in 10. The studies of measuring OD also reflect different approaches to these dimensions. Examining the developed scales shows that their dimensions are numerically different but shaped along similar structures. The scale that Weber and Unterrainer (2012) developed in Austria is based on the participatory dimension of OD. The ODS that Geçkil and Tikici (2015) developed in Turkey consists of five dimensions (participation-criticism, transparency, justice, equality, accountability). The ODS that Ahmed et al. (2019) developed in Pakistan consists of ten dimensions (freedom, fairness, integrity, tolerance, structure, shared responsibility, transparency, knowledge-sharing, accountability, learning environment). Similar to political democracy, cultural and historical differences, different perspectives on organizational democracy, and other social dynamics (religious, ethnicity, national) significantly shape organizational democracy.

Considering the contributions of business organizations to the socialization of political competencies and orientations, Pateman (1970) expresses the following spillover effect. Significant employee participation in democratic decision-making processes allows employees to experience political effectiveness. In the long run, experiencing political activity has an educational effect, promoting civic virtues, political participation, and active citizenship behaviors among employees not only in the workplace but also in civil society. Pircher-Verdorfer et al. (2012) state that democratic firms, which give their employees the opportunity to participate in tactical and strategic decision-making processes, are fractals of a democratic society and common welfare institutions. They include a field of socialization that supports employees in the (further) development of democratic competencies and orientations. We cannot completely separate organizational democracy from political democracy. Political democracy can cause many reflexes in individuals getting used to the principles of democracy, to accept it, to believe in its necessity, and to desire it in case of its absence.

This study is based on empirical studies of organizational democracy conducted in Turkey. For this reason, the reader may find useful a brief mention of Turkey's socio-political past and organizational democracy studies. Turkey shows the characteristics of a transition economy and society. One of the OECD countries, it is considered an emerging market. While economic institutions can sometimes create policies on their own, with economic priorities, unfortunately, we cannot say that they provide continuity in creating independent policies. Annual income per capita is below 10,000 $, and the economy has faced economic and currency crises (Gök and Kara, 2021) in different periods. Turkish democracy has a history of more than 200 years. The Charter of Alliance (Sened-i Ittifak, September 28, 1808) marks the beginning of Turkish democracy, as the first document that limited the authority of the Sultan (Lewis, 2007, p. 50). Despite the country's important political background in its geographical ground, it still lacks autonomous and established political and economic institutions and seems weak in the Western sense. For this reason, we could observe some authoritarian tendencies among elected officials in several periods. Despite the experience of more than 200 years of democracy, many interventions have hindered the maturation of political democracy. During the Ottoman period, the elected parliament was shut down on the Sultan's decision (February 14, 1878) and reconvened about 30 years later (July 23, 1908). In addition, in the 40 years period between 1960 and 2000, there were four military coups (1960, 1971, 1980, and 1997). Despite frequent interruptions, the democratic process has always got on track again. Huntington (1991) states that to qualify as an established democracy, a society must have changed its government through elections at least twice (p. 266–267). Turkey has met this test more than twice (Lewis, 2007, p. 28).

In his study on cultures, Hofstede (2021) finds that high power distance is normal in Turkish culture. Turkey scores high on power distance, meaning that the Turkish style is dependent and hierarchical, with generally inaccessible superiors. Turkish society is collectivist, implying that the “We” is significant; people are members of in-groups (families, clans, or organizations) that watch out for one another, in exchange for allegiance. The connection has a moral foundation, which always takes precedence over task completion. Hofstede (2021) points out that the Turkish society's masculinity score (45) is low. This means that softer components of society are cherished and fostered, such as leveling with others, consensus, and sympathy for the underdog. In both private and professional life, conflict avoidance, and reaching an agreement at the end are crucial. For Turks, leisure time is vital, when the entire family, clan, and friends get together to enjoy life. Hofstede (2021) states that the uncertainty avoidance score (85) is quite high in Turkish culture so there is a great need for laws and rules. He emphasizes that there is no dominant culture for long-term orientation and indulgence.

Despite Turkey's history of political democracy over more than two centuries, only a few studies on organizational democracy exist, but interest in OD has increased in recent years. An all-time search in the National Thesis Center (https://tez.yok.gov.tr/UlusalTezMerkezi/tarama.jsp) with the keyword “organizational democracy” returned 21 completed theses. The first study of OD in Turkey is a master's thesis completed in 2010 (Seker, 2010), which examines the level of adoption and implementation of organizational democracy in schools. Following this study, a scale to examine the organizational-democracy levels of academicians in a doctoral thesis appeared (Bozkurt, 2012). Developed for working with academicians, the scale consists of two subscales (participation and autonomy). However, no other study using this scale appeared in the literature. In addition, a review article (Erkal Coşan and Altin Gülova, 2014) appears to be the first study on organizational democracy published in Turkey. The number of studies on OD in Turkey has actually increased since 2014, and they mainly focus on determining employees' organizational democracy levels and associated factors.

The ODS (Organizational Democracy Scale), the focus of this study, measures the organizational-democracy level based on employee perceptions. Individuals make decisions on the basis of not only realities but also their perceptions of those realities. Perceiving is the process of giving meaning to the stimuli in the individual's environment. Our perceptions also create in our minds values, problems, and solutions for them. As perceptions vary among individuals, they can also vary for the same individual under different conditions. Therefore, differences between reality and perceived reality may exist. The concept of reality varies from region to region, from country to country, and even from person to person (Friman, 1999, p. 6). Employee perceptions of OD express the individual's “perceived reality.”

### Organizational Democracy Scale

The organizational democracy scale that Geçkil and Tikici (2015) developed is based on a seven-dimension theoretical construct. A result of their literature review to prepare the scale development was defining the conceptual structure of organizational democracy using seven dimensions (participation, criticism, transparency, justice, equality, accountability, and power-sharing).

During the scale-development process, one of these dimensions (criticism) was combined with another (participation), while a further construct (power-sharing) did not emerge as a separate dimension (Geçkil and Tikici, 2015). It seems acceptable that the criticism dimension should combine with the participation dimension. A reasonable criticism may emerge more prominently as a result of supporting participation. Uninformed criticism can occur in organizations where there are insufficient or no participatory practices. The inability to confirm the power-sharing dimension in the scale-development process represents a real loss. The literature emphasizes the importance of power-sharing for an established democracy at the organizational level (Kerr, 2004, p. 81). Kerr states that while power-sharing is attractive when it comes to state affairs, managers at the organizational level hesitate to share power, and the resistance at various management levels is an obstacle to the successful implementation of democratic processes. The organizational democracy scale's six dimensions are examined below.

Participation means involving employees in all decision-making processes, directly or through their representatives. Many researchers equate OD with participation and try to define it on that basis (Weber et al., 2008, 2009; Yazdani, 2010). Weber et al. (2009) define OD as employees' structurally supported participation in management. This kind of participation appears directly or representationally, continuously, in broad-based, institutionalized, and non-temporary or non-random ways. Through participation, employees become an element of decision processes and practices in matters concerning their work and can evaluate the results together (Geçkil, 2013).

Criticism reflects the evaluation of policies and procedures, work, and transactions, by employees and other stakeholders at all relevant levels, and the ability to freely express those evaluations. Thanks to criticism, mistakes do not persist. Some researchers consider criticism, also expressed as raising the employees' voice, the most important element of OD (Yazdani, 2010).

Transparency means openness in the administration, of great importance for the democratization of the administration (Üst Can, 2020). It represents not only the sharing of information but also the intention to share and the information's perceived quality. Transparency is among the ISO 26000 standards, required for OD (Hallström, 2010) since it is the availability, to every individual who participates in decision-making, of all information about transactions and actions in the organization. Also, the information must be accessible to members whom the transactions and actions affect (Forcadell, 2005).

Justice, or the concept of organizational justice, refers to distribution of gains (distributive justice), processes used in making distribution decisions (procedural justice), and inter-individual relations (interactional justice) (Gilliland and Chan, 2009). Organizational justice examines the perceptions of employees regarding the fairness of their treatment (Greenberg, 1990a). The main determinants of the perception of justice are how the added value that emerges as a result of organizational activity is shared, and what criteria guide promotions. OD requires fairness in income distribution. A steep income gap among individuals prevents the democratization of organizations and makes it difficult for democratic management principles to settle in the organization (Geçkil, 2013, p. 35).

Equality is everyone having the same rights and advantages. As an element of OD, it should not be accepted as mistaken for absolute equality. However, it should be equal treatment of those whose conditions are equal. Equality between the individuals should relate to such criteria as performance, education, seniority (Geçkil and Tikici, 2015). The essence of OD includes all employees receiving equal treatment and getting equal benefits (Ahmed et al., 2019).

Accountability has become an important practice recently; the public calls for managers to be more accountable. This means that the actions of any person or organization require a statement, defense, or obligation to an affected person or group (Messner, 2009; Eryilmaz and Biricikoglu, 2011). Kerr (2004) states that the most important principle distinguishing OD from other types of management is “accountability.” Unlike responsibility, accountability includes not only the ability to assume the consequences of actions but also explaining and defending the situation (Lindkvist and Llewellyn, 2003). It once represented only a concept relating to the field of accounting and finance, but after the 1980s, it began to apply to all kinds of managerial functions.

Since the ODS is published in Turkish, readers may find an explanation of the scale-development process useful. ODS is based on five point Likert-type response scales and encompasses 28 items and five subscales. The minimum score measuring employees' OD perceptions is 28, and the maximum is 140. Increasing total scores across all items and subscales reflect increased employee perception of OD. Interpretation of each subscale score is like that for the ODS total score (Geçkil and Tikici, 2015).

The scale-development process utilized a three-phase and ten-step model that Slavec and Drnovsek (2012) developed. To decide the items to include in the ODS, Geçkil and Tikici (2015) created a pool of 156 items, using the literature review and field experts. The researchers reviewed those items, deleted repetitive statements, and arrived at a 68-item draft scale. The candidate scale then went to the expert panel (11 faculty members from the relevant field) for an assessment of content validity. Items with a low Item Content Validity Index (I-CVI) were removed, and a 42-item candidate scale form resulted, with I-CVI values varying between 0.82 and 1.0. The Scale Content Validity Index (S-CVI) was calculated as 0.88. Polit and Beck (2006) recommend that the I-CVI value be higher than 0.78 and the S-CVI value higher than 0.80 (p. 491). Thus, the content validity of the scale was rated as good.

Thereafter, the candidate scale was applied to the sample of 438 people. The data were analyzed by mean, standard deviation, Pearson Moment Correlation, Cronbach‘s Alpha, Explanatory Factor Analysis, and Confirmatory Factor Analysis, using SPSS 21 and LISREL 8.8 programs. Exploratory Factor Analysis for the construct validity used the data of 285 people (42% female, 58% male, 56.1% academician, and 43.9% officer). Confirmatory Factor Analysis used data of 153 participants (65.2% female, 34.8% male; 13.6% physician, 53.7% nurse/midwife, 20.4% pharmacist, physiotherapist, laboratory worker, x-ray technician, and 12.2% medical secretary, computer technician).

As a result of the Exploratory Factor Analysis (EFA), a 28-item scale encompassing five factors emerged (Geçkil and Tikici, 2015, p. 60). The first factor, *Participation-Criticism*, consisted of 8 items, and the factor loads of the items varied between 0.71 and 0.42. This subscale included such items as “Managers encourage me to participate in organizational decisions,” and “Management takes criticism by employees into consideration.” The second dimension, *Transparency*, identified 6 items with factor loadings ranging from 0.76 to 0.54. The Transparency subscale included such statements as “The works are carried out according to principles of transparency in my organization,” and “There is an open and two-way communication in my organization.” The third dimension, *Justice*, consisted of 5 items with factor loadings ranging from 0.63 to 0.52. The Justice dimension encompassed such statements as “My organization has a fair reward system,” and “The wages and other incomes of the employees are determined by taking into account their contributions within their work and for their organization.” The fourth dimension, *Equality*, consisted of 6 items with factor loadings ranging from 0.70 to 0.47. This subscale included such items as “There is no gender discrimination in my organization,” and “Discrimination based on language, religion or race is not accepted in my organization.” The last dimension, *Accountability*, consisted of 3 items with factor loadings varying between 0.78 to 0.43. The Accountability subscale comprises items such as “Policies and procedures in our workplace can always be questioned by employees,” and “A culture of accountability has been developed in my institution” (Geçkil and Tikici, 2015, p. 61–62). The factor loads of the items in the ODS ranged from 0.42 to 0.78 (Geçkil and Tikici, 2015, p. 63). The cumulative variance of the ODS was 58.78%. The social sciences consider a variance rate in the range of 40%−60% sufficient (Scherer et al., 1988). Items 21 and 23 in the scale represent inverse statements and must be recoded.

The Cronbach's α reliability coefficient of ODS was α = 0.95 for the total ODS scale. For the following subscales, Cronbach's α amounted to *participation-criticism* α = *0.8*8, *transparency* α = 0.88, j*ustice* α = 0.80, *equality* α = 0.83, and *accountability* α = 74. The ODS scale was twice applied to a group of 45 people for test-retest within a 2-week interval. The test-retest correlation coefficient was 0.87 (*p* < 0.001). Confirmatory Factor Analysis was performed to confirm the model that emerged as a result of Exploratory Factor Analysis, and the findings suggested that the ODS scale had good fit values. The goodness fit indexes of the Organizational Democracy Scale with Confirmatory Factor Analysis are as follows: χ2 = 575.8, Df = 340, X^2^/df = 1.69, CFI = 0.97, and RMSEA = 0.064 (Geçkil and Tikici, 2015, p. 67). The original Turkish form of the organizational-democracy scale appears in Appendix A and Appendix B shows the form translated into English by the author.

For cross-validation purposes, the researcher assessed the convergence between the ODS and a similar scale. First, the similarities between the Organizational Justice Scale (OJS) and ODS subscales suggested that this convergence might exist. OJ is divided into two subscales: “fairness of results” (distributive justice) and “fairness of process” (procedural justice) (Gilliland and Chan, 2009, p. 169). In the 1980s, in addition to these two types, interpersonal relations were considered a new form of justice (interactional justice), recognized as a subcomponent of procedural justice in the 1990s (Cropanzano and Greenberg, 1997). The examination of the similarity between ODS and OJ considered distributive justice and procedural justice, in two investigative steps: first, the similarities between the subscales of ODS and OJS and, second, the effects of OD and OJ on several outcome variables in the field of individual and organizational psychology.

The “fairness of results subscale” (Gilliland and Chan, 2009) is similar to the justice, equality, and accountability dimensions of the ODS scale. While the perceptions of organizational outcomes and rewards and the equitable distribution of organizational assignments and promotions shape the justice subscale of the ODS, the equality subscale is attributable to distribution in accordance with regulations or directives. Perceptions of the fairness of the results can lead to subjective results relating to individual differences. The fact that the equality dimension proceeds on written rules can prevent the emergence of subjective differences. This feature reveals ODS's wider measurement nature than OJS's. On the other hand, the accountability dimension refers to the managers' accountability for the employees whom the work and operations affect. The perception of injustice regarding the results can cause negative employee behaviors toward superiors and the organization. The culture of accountability can prevent the emergence of negative behaviors, by endowing the employee with the power to solve problems.

Further, the fact that OD and OJ had similar effects on some typical outcome variables in the fields of individual and organizational psychology strengthened the idea of convergence between the two constructs. OJ turned out to be an important determinant of attitudes and behaviors. Employees perceiving their managers and organizations to be fair partly shape job satisfaction and organizational commitment (Martin and Bennett, 1996). Equality and fairness concerns drive decisions on remuneration and other resource allocations (Scarpello and Jones, 1996). Evidence shows that voluntary behavior in organizations, both positive organizational belonging behaviors and negative antisocial behaviors, have a significant association with perceptions of justice and fairness (Greenberg, 1990b; Moorman, 1991).

Likewise, the findings of the focused systematic review that will be presented in the sections Results and Discussion indicate that OD has positive effects on employees' job satisfaction and organizational commitment as well as on Organizational Citizenship Behaviors, organizational identification and work engagement. Further, the results of this review will show that OD significantly reduces individual and organizational consequences that lead to negative behaviors, including organizational depression, job stress level, and intention to quit the job. These results of cross-validation strongly confirmed our hypothesis of conceptual relationships between ODS and OJS.

Finally, the strong correlation between the two scales indicates that the selection of OJS is appropriate in terms of convergence. The Pearson correlation amounts to *r* = *0.8*0 (*p* < 0.001) between the ODS and the Turkish adaptation by Yildirim (2002) of the Organizational Justice Scale (OJS) that Niehoff and Moorman (1993) developed. The subscales of the ODS correlated with the overall OJS score as follows: Participant-Criticism *r* = 0.700 (*p* = 000), Transparency *r* = 0.702 (*p* = 000), Justice *r* = 0.671 (*p* = 000), Equality *r* = 0.692 (*p* = 000), Accountability *r* = 0.572 (*p* = 000).

After the development of the scale in Turkey and its introduction to the literature, researchers began to use ODS extensively. Authors have begun to reveal the effects of OD by examining its relationship to several variables relevant to organizational behavior and organizational structure. The findings of each of these studies are valuable on their own, but examining all of them together and revealing the similarities and differences between them, through a meta-analysis or a systematic review, can identify unique contributions to the literature. Such a systematic review or meta-analysis searching OD and related factors in Turkey is lacking; thus, this study intends to close that gap. In their meta-analytical study, Weber et al. (2020) examine the psychological and social consequences of employee participation in democratic enterprises. By contrast, this systematic review covers both participation and other dimensions of OD. In this respect, it can provide important contributions to the OD literature. Thus, this study aims to present to practitioners, policymakers, and scientists the core information that will form a basis for their further studies.

### The Aim and Questions of the Study

This focused systematic review aims to determine the OD levels of public- and private-sector employees in Turkey and their associated factors. The presentation of the study questions follows the PICOS format (P: Patient/Problem/ Population; I: Intervention; C: Comparators, O: Outcomes; S: Study design) (Petticrew and Roberts, 2006; Centre for Reviews and Dissemination, 2008), and the questions are:

1. What is the level of perceived OD of public- and private-sector employees in Turkey?
2. What are the potential outcomes (and additional correlates) associated with OD?

## Methods

### Article Type

This study is a focused systematic review of studies related to the OD scale in Turkey. This study establishes the systematic review protocol and the reporting of the articles in line with the PRISMA statement on systematic reviews (Page et al., 2021).

### Search Strategy

**Figure 1:**
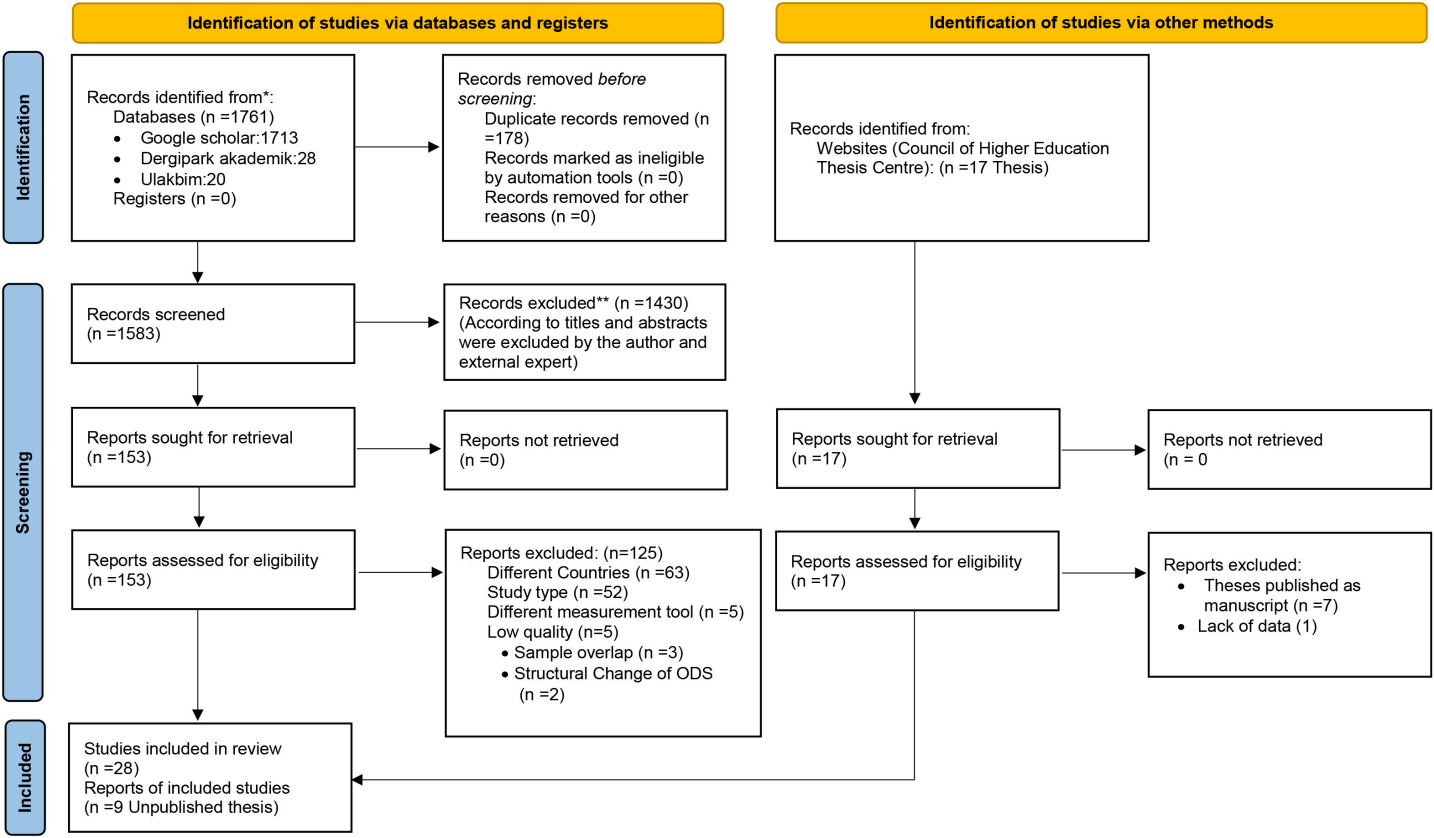
PRISMA 2020 flow diagram: Selection and inclusion process of studies for systematic reviews. *Consider, if feasible to do so, reporting the number of records identified from each database or register searched (rather than the total number across all databases/registers). **If automation tools were used, indicate how many records were excluded by a human and how many were excluded by automation tools. Adapted from Page et al. (2021).

The search included quantitative empirical studies using the ODS scale (Geçkil and Tikici, 2015) developed in Turkey. It was limited to literature published in journals, books, or congress books and unpublished national theses. The literature review covered the following steps:

- The following databases were searched for publications from January 2014, when the ODS was developed, to April 15, 2021, to find as many studies as possible that met the inclusion criteria. The last search was done on April 15, 2021. Using Turkish and English keywords, the search-engine databases used returned 1,767 records using Google Scholar (http://scholar.google.com.tr), the National Thesis Center (https://tez.yok.gov.tr/UlusalTezMerkezi/tarama.jsp), and Dergi Park (https://dergipark.org.tr/tr/), and Ulakbim (https://app.trdizin.gov.tr/advancedSearchs).
- To access additional publications, the researcher used personal contacts (i.e., authors who asked permission from the scale developer to use the scale in their research) and hand-searching (citations of the article reporting the ODS development and a search of conference papers). The references in the included publications were reviewed. In addition to the records identified through databases, the manual search produced a total of 11 publications.
- The Turkish keywords “örgütsel demokrasi” or “örgütsel demokrasi algisi” or “algilanan örgütsel demokrasi” or “örgütsel demokrasi ölçegi” were used for the search.
- The English keywords “organizational democracy” or “organizational democracy perception” or “perceived organizational democracy” or “organizational democracy scale” were searched.
- As a result of searching with these keywords, a total of 1,778 records (1,761 from the databases and 17 from the website of the Council of Higher Education Thesis Center) were obtained between January 2014 and April 2021. After examining these records, 178 duplicate studies were removed. The remaining 1,583 studies were examined by title and abstract, and 1,430 studies that the researcher considered irrelevant were excluded. Finally, 153 studies were analyzed as full text for the research. Of these, 63 were excluded because they were conducted in countries other than Turkey. Since 52 of these studies were books, book chapters, compilations, case studies, and scale-development studies, they were excluded because they did not represent quantitative or experimental studies. Five of the remaining studies were excluded because they used a tool that measured OD with different dimensions and content. Five more were excluded because of the quality assessment. Seven out of 17 theses were excluded because they were published as manuscripts already accessed through the database search. One thesis was excluded because of a lack of data. Nine unpublished theses were included. As a result, a total of 37 studies examining the OD perceptions of employees in Turkey were included in this systematic review (see Figure 1. PRISMA 2020 flow diagram).
- The total sample size of these 37 studies was *N* = 10,370 participants.

### Selection of Studies

Studies included in this systematic review qualified according to the inclusion and exclusion criteria.

**Inclusion criteria for the study**

- Quantitative empirical studies conducted in Turkey,
- Published in Turkish or English,
- Examining the level of OD in private and/or public institution employees,
- Examining the relationship between the level of OD and demographics, work characteristics.

**Exclusion criteria;**

- Quantitative or qualitative research review,
- A book or a book chapter that does not include an empirical study,
- Using a measurement tool other than the ODS (Geçkil and Tikici, 2015) to measure the level of OD.

### Evaluation of Methodological Quality of Studies

Making a methodological quality assessment to determine the possibility of bias in the design, conduct, and analysis stages of the studies to include in systematic reviews is recommended procedure (Moola et al., 2020). The researcher determined the methodological quality of 43 studies according to inclusion and exclusion criteria in this systematic review. The types of studies this systematic review includes are analytical and cross-sectional. For this reason, the quality-assessment tool used was the Analytical Cross-Sectional Studies Critical Appraisal Tool, developed by the Joanna Briggs Institute (JBI) and collaborators, and approved by the JBI Scientific Committee following extensive peer review (Moola et al., 2020). After the screening process examined the title, abstract and full text, 43 identified empirical studies were evaluated for quality, including 27 published articles, 10 unpublished theses (five of which were master's theses and five doctoral dissertations), five conference papers, and one working study. As a result of the quality evaluation, six studies were excluded due to sample overlap (*n* = 3), change in the factor structure of ODS (*n* = 2), and lack of data (*n* = 1). Appendix C shows the excluded studies. As a result of the quality assessment, this review included 37 studies. The master theses (Barutçu, 2019; Uysal, 2019; Yalçinkaya, 2019; Erdal, 2020; Kara, 2020) included in the present study were uploaded to a data repository (see https://osf.io).

### Data Extraction

The researcher prepared the data extraction form used to obtain the relevant data for the research review. It includes information concerning the publication year, type, language, sector type, sample size, participants' education level, ODS scores of participants, demographics, and variables whose relationship with OD is examined. It also includes the main results of these studies.

## Results

The results of this study, whose aim was to examine the perceived OD levels of employees and associated work and organizational psychological outcomes, appear in several basic tables. First, the means of the ODS total score and subscale scores of the employees are reported in Table 1. Then, Table 2 compares ODS total scores and subscale scores of private- and public-sector employees. Table 3 indicates descriptive characteristics of the 37 studies, including variables associated with OD, and some results. Further, Table 4 represents the main results in describing the correlation or regression coefficients of OD and its dimensions with related variables. The Appendices D and E delineate each study in more detail.

**Table 1:** Organizational democracy scale total and subscale scores of participants (*N* = 9,902).

	**Studies**	**Organizational democracy scale subscale and total scores**						
	**References**	***N***	**PC Mean ±SD**	**T Mean ±SD**	**J Mean ±SD**	**E Mean ±SD**	**A Mean ±SD**	**ODS total Mean ±SD**
1.	Kesen (2015a)^a^	142	3.87 ± 0.78	4.04 ± 0.76	3.82 ± 0.88	4.03 ± 0.69	3.80 ± 0.97	3.92 ± 0.79^*^
2.	Kesen (2015b)^a^	174	3.84 ± 0.74	3.88 ± 0.78	3.76 ± 0.88	4.05 ± 0.67	3.79 ± 0.96	3.87 ± 0.78^*^
3.	Geçkil et al. (2016)^c^	363	2.69 ± 0.85	3.05 ± 0.83	2.64 ± 0.87	3.14 ± 0.59	2.82 ± 0.93	2.87 ± 0.67
4.	Geckil and Tikici (2016)^b^	582	2.47 ± 0.83	2.70 ± 0.83	2.35 ± 0.87	2.86 ± 0.75	2.53 ± 0.97	2.59 ± 0.70
5.	Bakan et al. (2017)^a^	201	3.45 ± 0.77	3.62 ± 0.74	3.52 ± 0.77	3.62 ± 0.84	3.48 ± 0.88	3.54 ± 0.79^*^
6.	Geçkil et al. (2017)^b^	405	2.22 ± 0.91	2.60 ± 0.87	2.19 ± 0.87	3.02 ± 0.66	2.42 ± 0.98	2.49 ± 0.71
7.	Geçkil and Koçyigit (2017)^a^	144	3.16 ± 0.97	3.37 ± 1.01	3.10 ± 0.94	3.31 ± 0.70	3.23 ± 1.09	3.25 ± 0.80^**^
8.	Işik (2017)^b^	32	2.40 ± 0.93	3.08 ± 0.96	2.08 ± 0.86	3.23 ± 0.60	2.94 ± 1.15	2.75 ± 1.39
9.	Işikgöz et al. (2017)^b^	191	3.64 ± 1.01	3.72 ± 0.68	3.44 ± 0.67	3.84 ± 0.37	3.95 ± 1.26	3.70 ± 0.80
10.	Öge and Çiftçi (2017)^a^	77	3.02 ± 0.66	3.18 ± 0.72	2.95 ± 0.74	3.33 ± 0.54	3.11 ± 1.03	3.12 ± 0.49
11.	Atalay (2018)^b^	920	3.64 ± 0.90	3.70 ± 0.66	3.79 ± 0.78	3.30 ± 0.45	3.77 ± 0.84	3.62 ± 0.72^*^
12.	Aykanat and Yildiz (2018)^b^	120	2.72 ± 0.80	3.15 ± 0.1.01	2.76 ± 0.95	3.40 ± 0.81	3.15 ± 1.19	3.01 ± 0.92^*^
13.	Çankaya (2018)^b^	200	2.40 ± 1.05	2.46 ± 0.1.21	2.19 ± 1.11	2.96 ± 0.78	2.13 ± 1.11	2.42 ± 1.05
14.	Karagöz and Atilla (2018)^c^	142	3.28 ± 1.08	3.73 ± 0.96	3.33 ± 1.05	3.25 ± 1.26	3.22 ± 1.17	3.36 ± 1.11^**^
15.	Tokay and Eyüpoglu (2018)^a^	240	3.28 ± 0.85	3.63 ± 0.92	3.34 ± 0.84	3.21 ± 0.76	3.54 ± 0.92	3.40 ± 0.88
16.	Bakan and Gözükara (2019)^b^	181	2.80 ± 0.99	3.11 ± 1.01	2.68 ± 0.1.00	3.32 ± 0.97	2.87 ± 1.05	2.96 ± 1.00^**^
17.	Barutçu (2019)^a^	120	3.08 ± 0.95	3.12 ± 0.86	3.51 ± 0.83	3.61 ± 0.74	3.66 ± 0.76	3.34 ± 0.84^*^
18.	Günden (2019)^a^	367	3.24 ± 1.17	3.76 ± 1.03	3.21 ± 1.05	3.32 ± 1.19	3.11 ± 1.18	3.33 ± 1.12^*^
19.	Karatepe (2019)^c^	300	3.00 ± 0.84	3.21 ± 0.90	3.00 ± 1.00	3.20 ± 0.61	2.90 ± 0.97	3.08 ± 0.85^*^
20.	Naldöken and Limoncu (2019)^b^	326	2.51 ± 0.88	2.70 ± 0.91	2.19 ± 0.86	1.01 ± 0.84	2.52 ± 0.98	2.17 ± 0.70^**^
21.	Uysal (2019)^b^	316	3.85 ± 1.01	3.78 ± 1.03	3.91 ± 1.03	3.86 ± 1.02	3.80 ± 1.08	3.87 ± 0.1.03^*^
22.	Yalçinkaya (2019)^b^	397	3.08 ± 0.94	3.10 ± 0.87	3.54 ± 0.80	3.59 ± 0.74	3.70 ± 0.75	3.34 ± 0.84^*^
23.	Bilyay et al. (2020)^b^	202	3.28 ± 0.88	3.63 ± 0.94	3.06 ± 0.91	3.65 ± 0.88	3.12 ± 1.01	3.29 ± 0.72
24.	Erdal (2020)^b^	345	2.86 ± 1.16	3.19 ± 0.1.16	2.66 ± 0.1.19	3.20 ± 0.1.25	2.86 ± 0.1.12	2.95 ± 1.18^*^
25.	Erkasap (2020)^a^	509	3.14 ± 0.94	3.37 ± 0.95	3.04 ± 0.89	3.80 ± 0.86	3.25 ± 0.92	3.33 ± 0.72
26.	Erkasap and Ülgen (2020)^a^	225	3.12 ± 0.95	3.34 ± 0.96	3.07 ± 0.91	3.76 ± 0.84	3.23 ± 0.95	3.31 ± 0.73^**^
27.	Kara (2020)^a^	300	3.04 ± 0.99	3.48 ± 0.81	3.30 ± 0.85	3.58 ± 0.59	4.02 ± 0.77	3.40 ± 0.82^*^
28.	Karadağ and Geçkil (2020)^b^	192	2.87 ± 0.82	3.14 ± 0.91	2.74 ± 0.88	3.21 ± 0.81	2.71 ± 0.92	2.93 ± 0.76
29.	Pelenk (2020)^a^	380	4.10 ± 0.70	4.18 ± 0.58	4.16 ± 0.68	4.18 ± 0.52	4.27 ± 0.67	4.18 ± 0.63
30.	Üst Can (2020)^c^	281	3.01 ± 1.01	3.12 ± 0.98	2.81 ± 0.1.03	3.22 ± 0.60	3.03 ± 0.83	3.04 ± 0.89
31.	Yildirim and Deniz (2020)^a^	252	2.88 ± 0.93	3.33 ± 0.92	2.69 ± 1.03	Removed	3.01 ± 1.01	2.98 ± 0.84
32.	Çavuş and Biçer (2021)^a^	257	3.42 ± 0.90	3.52 ± 0.99	3.20 ± 0.1.07	Removed	3.18 ± 0.1.08	3.36 ± 0.99^*^
33.	Geçkil and Şendoğdu (2021)^c^	397	3.09 ± 0.92	3.43 ± 0.86	3.07 ± 0.93	3.37 ± 0.63	3.21 ± 0.94	3.24 ± 0.74
34.	Tokgöz and Önen (2021)^b^	622	3.80 ± 0.80	4.25 ± 0.66	3.75 ± 0.90	4.05 ± 0.67	3.71 ± 0.90	3.90 ± 0.65^**^
35.	Bilge et al. (2020)^a^	209						No Data^***^
36.	Can and Dogan (2020)^b^	129						3.42 ± 1.04^***^
37.	Senol and Aktaş (2017)^a^	130						2.95 ± 0.89^***^
^a^Private Sector	3,727	3.34 ± 0.90	3.59 ± 0.87	3.33 ± 0.89	3.68 ± 0.78	3.49 ± 0.93	3.47 ± 0.82
^b^Public Sector	5,160	3.10 ± 0.92	3.31 ± 0.85	3.08 ± 0.90	3.25 ± 0.74	3.15 ± 0.96	3.17 ± 0.80
^c^Mix(Public and Private)	1,483	2.98 ± 0.92	3.26 ± 0.89	2.93 ± 0.96	3.24 ± 0.67	3.02 ± 0.94	3.09 ± 0.81
Total	10,370	3.18 ± 0.91	3.42 ± 0.86	3.17 ± 0.91	3.47 ± 0.74	3.27 ± 0.95	3.30 ± 0.81

a *Private Sector,*

b *Public Sector,*

c *Public and Private Sector.*

*PC, Participate-Criticism; T, Transparency; J, Justice; E, Equality; A, Accountability.*

* *The mean of the ODS total score was not given in the study, so the weighted mean of the ODS total score was calculated by the subscales or items summing.*

** *These data are unpublished data obtained from the authors.*

*** *Studies with different scale structures*.

**Table 2:** Comparison of ODS and subscale scores of private and public sector employees.

**ODS total and subscales**	**Sector type**	***N***	**Mean rank**	**Sum of ranks**	**Mann-Whitney U**	***Z***	***P***
Participation-Criticism	Private	14	18.43	258.00	57.000	−2.096	0.036
	Public	15	11.80	177.00			
Transparency	Private	14	18.32	256.50	58.500	−2.030	0.042
	Public	15	11.90	178.50			
Justice	Private	14	18.14	254.00	61.000	−1.921	0.055
	Public	15	12.07	181.00			
Equality	Private	14	17.38	208.50	49.500	−1.977	0.048
	Public	15	11.30	169.50			
Accountability	Private	14	18.25	255.50	59.500	−1.987	0.047
	Public	15	11.97	179.50			
ODS total	Private	14	18.50	259.00	56.000	−2.140	0.032
	Public	15	11.73	176.00			

**Table 3:** The characteristics of the studies included in focused systematic review.

**Number of studies**	**Type of article and publication language**	**Publication language**	**Sector type**	**Sample size**	**Educational status**	**Cronbach's alpha reliability coefficient**	**Variables searched to be related**
37	Manuscript: 25 (67.6%) Unpublished Thesis: 9 (24%) Conference Paper: 2 (5.4%) Working Paper: 1 (3%)	Turkish: 27 (73%) English: 10 (27%)	Public: 16 (43.2%) Private: 16 (43.2%) Public and Private (Mixed): 5 (13.6%) **Sector:** Health: 7 (18.9%) Education: 7 (18.9%) Banking: 4 (10.8%) Hospitality: 2 (5.4 %) Retail: 2 (5.4 %) Various: 3 (8.1 %) Other: 12 (32.5%)	*N* = 10,370 Female: 4,864 (46.9%) Male: 5,128 (49.5%) Unknown: 378 (3.6%)	Primary school+ High school: 1,781 (17.1%) Graduate+ Postgraduate: 6,949 (67%) Unknown: 1,653 (15.9%)	0.70–0.79: 40.80–0.89: 3 Over 0.90: 20 Subscales: 7 Participate-Criticism: 0.805–0.938 Transparency: 0.718–0.924 Justice: 0.712–0.889 Equality: 0.660–0.916 Accountability: 0.679–0.850	•Demographics: 20 (54.1%) •Pcychological Variables (*n* = 21) - Organizational citizenship behaviors (OCB): 5 (13.5%) - Organizational dissent: 5 (13.5%) - Job satisfaction: 3 (8.1%) - Psychological capital: 3 (8.1%) - Organizational commitment: 3 (8.1%) - Organizational silence: 3 (8.1%) - Organizational identification: 2 - Social capital: 1 - Intrapreneurship tendency:1 - Organizational justice:1 - Organizational support:1 - Political sensitivity:1 - Organizational depression:1 - Quality of work life: 1 - Organizational culture: 1 - Job stress level: 1 - Employee performance:1 - Work engagement: 1 - Intention to quit from job: 1 - Ethical leadership: 1 - Psychological empowerment: 1

**Table 4:** The correlations and regression of related factors with ODS total and subscales.

**Related factors**		**ODS total**	**ODS subscales**				
			**Participation-Critism**	**Transparency**	**Justice**	**Equality**	**Accountability**
		***r/β/R* ^2^**	**r/β**	**r/β**	**r/β**	**r/β**	**r/β**
Geckil and Tikici (2016)	**OCB total**	*p >* 0.05	*p >* 0.05	*p >* 0.05	*p >* 0.05	*p >* 0.05	*p >* 0.05
	Altruism	−0.186^**^	−0.167^**^	−0.152^**^	−0.193^**^	−0.118^*^	−0.137^*^
	Conscientiousness	0.579^**^	0.486^**^	0.682^**^	0.407^**^	0.404^**^	0.379^**^
	Courtesy	0.786^**^	0.704^**^	0.759	0.714^**^	0.470^**^	0.577^**^
	Sportsmanship	*p >* 0.05	*p >* 0.05	*p >* 0.05	*p >* 0.05	*p >* 0.05	*p >* 0.05
	Civic virtue	0.892^**^	0.862^**^	0.857^**^	0.732^**^	0.531^**^	0.638^**^
Tokay and Eyüpoglu (2018)	**OCB total**	0.257^**^	0.245^**^	0.201^**^	0.329^**^	0.404^**^	0.309^**^
Barutçu (2019)	**OCB total**	*R*^2^ = 0.741; *p =* 0.000	*P >* 0.5	β=0.074; p:0.030	*β =* 0.454; p:0.000	*β =* 0.501; p:0.000	*β =* 0.105; p:0.008
Günden (2019)	**OCB total**	0.40^*^					
Çavuş and Biçer (2021)	**OCB total**						
	Altruism		0.431^**^	0.357^**^	0.369^**^		0.364^**^
	Conscientiousness		0.496^**^	0.442^**^	0.438^**^		0.435^**^
	Courtesy		−0.458^**^	−0.390^**^	−0.468^**^		−0.424^**^
	Sportsmanship		0.432^**^	0.414^**^	0.454^**^		0.487^**^
	Civic virtue		0.676^**^	0.620^**^	0.674^**^		0.614^**^
Bilyay et al. (2020)	**Organizational dissent**	0.220^*^					
	Upward dissent	0.480^*^					
	Lateral dissent	*p >* 0.05					
Erdal (2020)	**Organizational dissent**	0.647					
Erkasap (2020)	**Organizational dissent**	β = 0.304; *p =* 0.003					
	Upward dissent	*p >* 0.05	*p >* 0.05	β = 0.464; *p =* 0.001	β = −0.451; *p =* 0.001	*p >* 0.05	β = −0.332; *p =* 0.001
	Lateral dissent		β = 0.336; *p =* 0.001	β = −0.286; *p =* 0.001	*p >* 0.05	β = −0.205; *p =* 0.001	*p >* 0.05
Erkasap and Ülgen (2020)	**Organizational dissent**		*p >* 0.05	β = 0.286; *p =* 0.001	β = −0.251; *p =* 0.001	β = −0.150; *p =* 0.020	β = −0.228; *p =* 0.001
	Upward dissent		*p >* 0.05	β = 0.459; *p =* 0.001	β = −0.445; *p =* 0.001	*p >* 0.05	β = −0.331; *p =* 0.001
	Lateral dissent		β = 0.267; *p =* 0.001	β = −0.192; *p =* 0.001	*p >* 0.05	β = −0.262; *p =* 0.001	*p >* 0.05
Pelenk (2020)	**Organizational dissent**						
	Upward dissent		*p >* 0.05	*p >* 0.05	*p >* 0.05	*p >* 0.05	*p >* 0.05
	Lateral dissent		0.263^**^	0.258^**^	0.216^*^	*p >* 0.05	*p >* 0.05
Kesen (2015a)	**Job satisfaction**		0.372^**^	0.397^**^	0.495^**^	0.438^**^	0.237^**^
Geçkil et al. (2017)	**Job satisfaction**	0.622^**^	0.536^**^	0.566^**^	0.527^**^	0.494^**^	0.453^**^
	Intrinsic satisfaction	0.538^**^	0.467^**^	0.503^**^	0.415^**^	0.449^**^	0.391^**^
	Extrinsic satisfaction	0.613^**^	0.525^**^	0.546^**^	0.559^**^	0.468^**^	0.447^**^
Çankaya, 2018	**Job satisfaction**		0.475^**^	0.547^**^	0.563^**^	0.449^**^	0.517^**^
	Intrinsic satisfaction		0.419^**^	0.480^**^	0.498^**^	0.411^**^	0.473^**^
	Extrinsic satisfaction		0.510^**^	0.590^**^	0.602^**^	0.457^**^	0.530^**^
Geçkil et al. (2016)	**Psychological capital**	0.126^*^	0.110^*^	0.174^**^	*p >* 0.05	*p >* 0.05	0.153^**^
	Optimism	0.115^*^	0.110^*^	0.137^**^	*p >* 0.05	*p >* 0.05	0.185^**^
	Resilience	0.118^*^	*p >* 0.05	0.164^**^	*p >* 0.05	*p >* 0.05	0.141^**^
	Hope	*p >* 0.05	*p >* 0.05	0.150^**^	*p >* 0.05	*p >* 0.05	0.123^*^
	Self–efficacy	0.106^*^	0.121^*^	0.153^**^	*p >* 0.05	*p >* 0.05	*p >* 0.05
Geçkil and Koçyigit (2017)	**Psychological capital**	0.338^**^	0.292^**^	0.274^**^	0.264^**^	0.349^**^	0.275^**^
	Optimism	*p >* 0.05	*p >* 0.05	*p >* 0.05	*p >* 0.05	*p >* 0.05	*p >* 0.05
	Resilience	0.561^**^	0.519^**^	0.477^**^	0.471^**^	0.500^**^	0.389^**^
	Hope	0.301^**^	0.254^**^	0.246^**^	0.227^*^	0.301^**^	0.276^**^
	Self–efficacy	0.268^**^	0.237^**^	0.207^*^	0.196^*^	0.270^**^	0.250^**^
Karagöz and Atilla (2018)	**Psychological capital**	0.391^**^					
Naldöken and Limoncu (2019)	**Organizational commitment**	0.427^**^	0.336^**^	0.302^**^	0.351^**^	0.383^**^	0.446^**^
	Affective commitment	0.368^**^	0.321^**^	0.270^**^	0.304^**^	0.296^**^	0.330^**^
	Continuance commitment	*p >* 0.05	*p >* 0.05	*p >* 0.05	*p >* 0.05	*p >* 0.05	*p >* 0.05
	Normative commitment	0.489^**^	0.401^**^	0.352^**^	0.416^**^	0.415^**^	0.470^**^
Uysal (2019)	**Organizational commitment**	*p <* 0.05
Yalçinkaya (2019)	**Organizational commitment**	*R*^2^ = 0.126; p:0.000					
	Affective commitment		*p >* 0.05	*p >* 0.05	*p >* 0.05	β = 0.225; p:0.015	*p >* 0.05
	Continuance commitment		*p >* 0.05	*p >* 0.05	*p >* 0.05	*p >* 0.05	*p >* 0.05
	Normative commitment		β = −0.376; p:0.000	β = 0.482; p:0.000	*p >* 0.05	*p >* 0.05	*p >* 0.05
Erkasap (2020)	**Organizational silence**	β = −0.309; p:0.004					
	Acquiescent silence		β = −0.274; p:0.001	β = −0.310; p:0.001	β = −0.134; p:0.023	*p >* 0.05	*p >* 0.05
	Defensive silence		*p >* 0.05	*p >* 0.05	β = −0.305; p:0.001	β = −0.162; p:0.004	*p >* 0.05
	Pro-social silence		*p >* 0.05	*p >* 0.05	*p >* 0.05	*p >* 0.05	β=0.151 p:0.001
Karadağ and Geçkil (2020)	**Organizational silence**	−0.218^**^	−0.182^*^	−0.165^*^	−0.159^*^	−0.192^**^	−0.258^**^
	Acquiescent silence	−0.192^**^	−0.185^*^	−0.165^*^	*p >* 0.05	−0.156^*^	−0.218^**^
	Defensive silence	*p >* 0.05	0.149^*^	*p >* 0.05	*p >* 0.05	*p >* 0.05	*p >* 0.05
	Pro–social silence	−0.293^**^	−0.283^**^	−0.255^**^	−0.184^*^	−0.293^**^	−0.279^**^
Kesen (2015a)	**Organizational identification**		0.433^**^	0.383^**^	0.417^**^	0.381^**^	0.304^**^
Kesen (2015b)	**Organizational identification**		0.482^**^	0.472^**^	0.375^**^	0.399^**^	0.415^**^
Aykanat and Yildiz (2018)	**Social capital**						
	Structural		0.567^**^	0.556^**^	0.621^**^	0.600^**^	0.645^**^
	Relational		0.303^**^	0.327^**^	0.338^**^	0.591^**^	0.507^**^
	Cognitive		0.503^**^	0.516^**^	0.460^**^	0.688^**^	0.658^**^
Öge and Çiftçi (2017)	**Intrapreneurship tendency**	0.668^**^					
	Innovation	0.521^**^	0.402^**^	0.461^**^	*p >* 0.05	0.515^**^	0.267^*^
	Risk-taking and proactivity	0.642^**^	0.663^**^	0.544^**^	0.268^*^	*p >* 0.05	0.419^**^
	Autonomy	0.353^**^	0.471^**^	0.291^*^	*p >* 0.05	*p >* 0.05	0.324^**^
Bakan et al. (2017)	**Organizational justice**						
	Distributive		0.545^**^	0.553^**^	0.595^**^	0.503^**^	0.513^**^
	Procedural		0.547^**^	0.628^**^	0.592^**^	0.575^**^	0.557^**^
	Interpersonal		0.479^**^	0.549^**^	0.523^**^	0.555^**^	0.478^**^
	Informational		0.498^**^	0.606^**^	0.510^**^	0.553^**^	0.769^**^
Bakan et al. (2017)	**Organizational support**		0.403^**^	0.476^**^	0.365^**^	0.412^**^	0.714^**^
Karatepe (2019)	**Political sensitivity**	0.256^**^	0.191^**^	0.259^**^	0.266^**^	0.193^**^	0.172^**^
	Knowledge	0.285^**^	0.208^**^	0.269^**^	0.317^**^	0.187^**^	0.254^**^
	Cognition	0.166^*^	0.130^*^	0.199^*^	*p >* 0.05	0.169^*^	0.144^*^
	Participation	0.136^*^	0.119^*^	0.145^*^	0.178^*^	0.202^**^	0.140^*^
	Interest	0.150^**^	0.136^*^	0.139^*^	0.195^**^	0.158^*^	0.147^*^
Bakan and Gözükara (2019)	**Organizational depression**		−0.601^**^	−0.714^**^	−0.602^**^	−0.577^**^	−0.620^**^
Geçkil and Şendoğdu (2021)	**Quality of work life**	0.801^**^	0.771^**^	0.776^**^	0.726^**^	0.545^**^	0.600^**^
	Job and career satisfaction	0.676^**^	0.628^**^	0.652^**^	0.612^**^	0.451^**^	0.539^**^
	General wellbeing	0.716^**^	0.693^**^	0.698^**^	0.634^**^	0.509^**^	0.531^**^
	Control at work	0.684^**^	0.697^**^	0.635^**^	0.624^**^	0.410^**^	0.534^**^
	Working conditions	0.745^**^	0.700^**^	0.738^**^	0.677^**^	0.527^**^	0.547^**^
	Stress at work	0.516^**^	0.463^**^	0.521^**^	0.465^**^	0.421^**^	0.359^**^
	Home-work interface	0.732^**^	0.735^**^	0.705^**^	0.676^**^	0.459^**^	0.539^**^
Pelenk (2020)	**Organizational culture**						
	Clan		−0.108^*^	−0.105^*^	−0.120^*^	*p >* 0.05	*p >* 0.05
	Adhocracy		0.103^*^	*p >* 0.05	*p >* 0.05	*p >* 0.05	*p >* 0.05
	Market		0.166^**^	0.276^**^	0.223^**^	0.283^**^	0.231^*^
	Hierarchical		0.175^**^	*p >* 0.05	*p >* 0.05	0.275^**^	0.219^*^
Tokgöz and Önen (2021)	**Job stress level**		*p >* 0.05	*p >* 0.05	β = −0.14; p:0.01	β = −0.24; p:0.001	β = 0.14; p:0.001
Kesen (2015b)	**Employee performance**		0.353^**^	0.362^**^	0.308^**^	0.330^**^	0.272^**^
Yildirim and Deniz (2020)	**Work engagement**	β = 0.407; p:0.001					
	Vigor		β = −0.294; *p <* 0.001	β= 0.855 *p <* 0.001	β= −0.288 *p <* 0.001		β= −0.075 p:0.035
	Dedication		β= −0.343 *p <* 0.001	β= 0.867 *p <* 0.001	β= −0.215 *p <* 0.001		β= −0.073 p:0.036
	Absorption		β= −0.205 *p <* 0.001	β= 0.746 *p <* 0.001	β= −0.402 *p <* 0.001		*p >* 0.05
Kara (2020)	**Intention to quit from job**	−0.418^**^	−0.389^**^	−0.417^**^	−0.476^**^	−0.165^**^	*p >* 0.05

* *p <0.01,*

** *p <0.001.*

*–There is no reported data for blank cells in the table*.

Table 1 encompasses ODS total and subscale scores (*N* = 9,902 participants) from the 37 studies included in this focused systematic review. Table 1 shows the calculated weighted averages of the ODS total scores of the employees (mean = 3.30 ± 0.81). The results of the subscale scores indicate that the employees show the lowest score with regard to the justice subscale (mean = 3.17 ± 0.91), and the highest score with regard to the equality subscale (mean = 3.47 ± 0.74). The private-sector employees' ODS total score (*U* = 56.000; *p* = 0.032), participation-criticism score (*U* = 57.000; *p* = 0.036), transparency score (*U* = 58.500; *p* = 0.042), equality score (*U* = 49.500; *p* = 0.048), and accountability score (*U* = 59.500; *p* = 0.047) were significantly higher than the scores of public-sector employees (Table 2). There was no significant difference between the justice subscale scores of private- and public-sector employees (*U* = 61.000; *p* = 0.055).

Table 3 indicates that 25 (67.6 %) of the 37 included studies represent published manuscripts, and 9 (24%) of them are unpublished thesis. Most of the studies (73%) are written in Turkish and 27% are in English. The sample of this systematic review consists of *N* = 10,370 participants, half of whom (49.5%) were male. The venues of the studies include 43.2% in public-sector organizations and 43.2% in private-sector enterprises. Seven (18.9%) of the studies were conducted in the health sector, 7 (18.9%) in the education sector, and 4 (10.8%) in the banking sector. The professional education level of the majority of the employees (67%) was “university graduate” or higher.

A significant relationship between age and ODS scores occurred in 6 of 11 studies (Geçkil et al., 2016, 2017; Çankaya, 2018; Kara, 2020; Üst Can, 2020; Geçkil and Şendoğdu, 2021; see Appendix D). In 4 of these studies, the ODS scores of the participants over the age of 40 were significantly higher than those of participants under the age of 40 (Geçkil et al., 2016, 2017; Çankaya, 2018; Kara, 2020). In one study, the ODS score of those under the age of 31 was higher than the score of those between the ages of 32 and 37 (Geçkil and Şendoğdu, 2021), while in another study, a negative significant but weak correlation was found between ODS accountability and age (Üst Can, 2020). In another 5 studies, no significant association between age and ODS appeared (Geckil and Tikici, 2016; Tokay and Eyüpoglu, 2018; Barutçu, 2019; Yalçinkaya, 2019; Erdal, 2020).

Fifteen studies investigated the relationship between gender and ODS scores (see Appendix D). A significant relationship between gender and ODS scores appeared in a total of 7 studies. In 6 of these, men's ODS scores were significantly higher (Geckil and Tikici, 2016; Geçkil et al., 2017; Çankaya, 2018; Karatepe, 2019; Yalçinkaya, 2019; Kara, 2020). In one study, female participants' scores were higher (Karadağ and Geçkil, 2020). Eight studies showed no significant correlation between gender and ODS scores (Işikgöz et al., 2017; Tokay and Eyüpoglu, 2018; Barutçu, 2019; Naldöken and Limoncu, 2019; Bilyay et al., 2020; Erdal, 2020; Üst Can, 2020; Geçkil and Şendoğdu, 2021).

Eleven studies investigated the relationship between education levels and ODS scores. In 8 of these, it was reported that there was no significant relationship between both variables (Geckil and Tikici, 2016; Geçkil et al., 2016; Işikgöz et al., 2017; Tokay and Eyüpoglu, 2018; Barutçu, 2019; Yalçinkaya, 2019; Geçkil and Şendoğdu, 2021). Three studies indicated a negative correlation between the two variables (Karatepe, 2019; Kara, 2020; Üst Can, 2020). On the contrary, in one study, scores of those employees with vocational school degrees were higher than those with high school degrees (Çankaya, 2018).

Appendix D further shows the findings of 11 studies that examined the relationship between the ODS and the marital status of the participants. In 6 of these studies, no significant relationship between the marital status of the participants and their ODS scores appeared (Işikgöz et al., 2017; Tokay and Eyüpoglu, 2018; Barutçu, 2019; Yalçinkaya, 2019; Erdal, 2020; Üst Can, 2020). ODS scores of singles in 4 studies (Çankaya, 2018; Karatepe, 2019; Geçkil and Şendoğdu, 2021; Naldöken and Limoncu) and of married participants in one study were significantly higher (Kara, 2020).

Sixteen studies examined the relationship between participants' job tenure and ODS. Eight of them indicate no significant relationship between those variables (Geçkil et al., 2016; Tokay and Eyüpoglu, 2018; Barutçu, 2019; Naldöken and Limoncu, 2019; Yalçinkaya, 2019; Bilyay et al., 2020; Erdal, 2020; Üst Can, 2020). A significant relationship between the duration of job experience and ODS scores appeared in eight studies. In four of them, those participants with <5 years of experience had higher ODS scores (Geckil and Tikici, 2016; Geçkil et al., 2017; Işikgöz et al., 2017; Geçkil and Şendoğdu, 2021); in three of them, participants with longer experience had higher ODS scores (Çankaya, 2018; Karatepe, 2019; Kara, 2020).

The other 21 variables, whose relationships with ODS the studies in this focused systematic review examine, represent typical work and organizational psychological outcomes or correlates of OD (for details see Table 3). The findings that Appendix D presents and Table 4 summarizes relate to employees' behavior, experience, and attitudes. Following the approach of Positive Organizational Behavior (Dutton and Glynn, 2007; Campbell Quick et al., 2010), several outcome variables represent positive organization and employee effects (e.g., organizational citizenship behaviors, job satisfaction, psychological capital, organizational commitment). Organizational dissent, silence, and depression, as well as job stress and intention to quit the job, are considered negative outcomes of low levels of organizational conditions (e.g., Weber et al., 2020).

Four of the studies included in this systematic review (Işik, 2017; Işikgöz et al., 2017; Atalay, 2018; Üst Can, 2020) do not appear in Table 4 because they examined only the relationship between ODS and demographic variables. In Table 4, high regression and correlation coefficients between some variables and ODS subscales draw attention. For example, Yildirim and Deniz (2020) report very high beta coefficients between work engagement and the ODS subscale “transparency” (ß = 0.746 to 0.867). Geckil and Tikici (2016) found very high correlations between the OCB civic virtue subscale and ODS and its subscales (*r* = 531–892). This situation may raise the question of whether those items are so similar that they are measuring the same phenomena. However, a comparison of the item contents of these subscales showed such different semantic contents that they do not represent the same thing.

Both theory and existing empirical research let assume that features of OD will be positively associated with features of OCB (see the meta-analysis by Weber et al., 2020). This may be the case because collective planning and decision making allows as well as requires mutual help among the participating employees. OCB refers to constructive and responsible participation in organizational processes (Organ, 1988). Five studies (13.5%) investigated the relationship between ODS and organizational citizenship behaviors (OCB). Statistically significant positive relationships between total scores of ODS and OCB appeared in three studies (Tokay and Eyüpoglu, 2018; Barutçu, 2019; Günden, 2019). In one study, the ODS total score was associated (Tokay and Eyüpoglu, 2018), in another study it was not associated with OCB total (Geckil and Tikici, 2016). Two studies (Geckil and Tikici, 2016; Çavuş and Biçer, 2021) examined relationships between scores of ODS subscales and OCB subscales. In one study (Geckil and Tikici, 2016), no significant relationship between ODS and its subscales and the OCB sportsmanship subscale was found. In the same study, and not in line with OD theory, a negative and weak correlation (*r* = −0.118 to −0.193) was identified between ODS total and its subscales and the OCB altruism subscale. ODS total and all ODS subscales correlated significantly and positively with the OCB conscientiousness, courtesy, and civic virtue subscales (*r* = 0.379 to 0.892; Geckil and Tikici, 2016). Despite a negative association between four of the ODS subscales and OCB courtesy, positive relationships (*r* = 0.414–0.487) were found between ODS subscales and OCB altruism, conscientiousness, sportsmanship, and civic virtue (Çavuş and Biçer, 2021). A further study demonstrated positive associations between ODS total, together with the transparency, justice, equality, and accountability ODS subscales and OCB total, but no relationship was found between the ODS participation-criticism subscale and OCB total (Barutçu, 2019).

Organizational dissent means that employees can express their discomfort and ideas within the organization. Organizational dissent is described as a “necessary devil” in modern organizations (Zeng, 2018). Offering work-related freedom of thought, OD can lead to the emergence and existence of organizational dissent. A democratic work environment leads employees to embrace work-related facts and, thus, increase their performance (Ahmed et al., 2019). This review includes 5 (13.5%) studies that explore the relationship between ODS and organizational dissent. Three report positive and significant associations between ODS total and organizational dissent total scores. The level of the relationship was weak (*r* = 0.220) in one study (Bilyay et al., 2020) and moderate or strong (respectively, β = 0.304; *r* = 0.647) in two other studies (Erdal, 2020; Erkasap, 2020) (see Appendix D and Table 4). Two studies investigated the relationship between ODS total and organizational upward dissent. One showed no relationship (*p* > 0.5) (Erkasap, 2020) while a moderately positive relationship was found in the other (*r* = 0.480; *p* < 0.5) (Bilyay et al., 2020). The relationship between ODS total and organizational lateral dissent was investigated in only one study, no significant correlation appeared. The studies by Erkasap (2020) and Erkasap and Ülgen (2020) revealed a positive, significant, and moderate relationship between the ODS transparency subscale and upward organizational dissent. In two studies, negative, significant, and moderate relationships were found between the ODS justice and accountability subscales and the organizational dissent total (Erkasap, 2020; Erkasap and Ülgen, 2020). Similarly, the study of Erkasap and Ülgen (2020) found negative correlations between the ODS justice and accountability subscales and upward dissent. Further, in two studies, ODS transparency and equality subscales had weak and negative effects on lateral dissent (Erkasap, 2020; Erkasap and Ülgen, 2020), whereas ODS transparency had a positive influence on the latter and equality had none in Pelenk's 2020 study. In three studies, ODS participation-criticism affected lateral dissent significantly positively (Erkasap, 2020; Erkasap and Ülgen, 2020; Pelenk, 2020), thought, contrary to theoretical assumptions, no significant associations were identified with upward dissent.

It is widely believed that the employee participation may affect employee's job satisfaction, because, for example, participation satisfies employees' basic needs for autonomy and competence. Therefore, employee participation seems to be an important determinant of job satisfaction (Heller et al., 1998; Bhatti and Qureshi, 2007). As Appendix D and Table 4 show that positive, significant, and moderate correlations were found in all 3 relevant studies (8.1%) that examined the relationship between the ODS subscales and job satisfaction subscales (*r* = 0.237–0.602) (Kesen, 2015a; Geçkil et al., 2017; Çankaya, 2018). One study examined the associations between ODS total and job satisfaction and its subscales, which revealed significant and strong positive correlations (*r* = 0.538–0.622) (Geçkil et al., 2017).

Employees' belief in their own abilities, strong will, having a positive perspective, and being able to make positive changes in failure or distress are closely related to their psychological capital (Luthans and Youssef, 2004). Organizational democracy can improve the positive mood of the employees by changing the socio-moral atmosphere of organizations. In turn, positive mood of employees can increase their optimism, hope, resilience and self-efficacy. However, it is thought that the change in psychological capital may be related to individual factors rather than the environment. Three studies (8.1%) inspected the relationship between ODS and psychological capital. In one (Geçkil et al., 2016), a weak correlation (*r* = 0.126) and in the other two (Geçkil and Koçyigit, 2017; Karagöz and Atilla, 2018), a moderate correlation (*r* = 0.338–0.391) appeared between the total scores of ODS and psychological capital. Not in line with theory, Geçkil et al. (2016) found no significant relationship between ODS total and hope; further, Geçkil and Koçyigit (2017) found no relation between ODS total, ODS subscales and optimism. These results, which are inconsistent with OD theory, are thought to be related to individual factors. In one study, ODS total and some of the ODS subscales were associated significantly and positively with the total score and some subscales of psychological capital, namely, resilience, hope, and self-efficiency. Weak correlations were found between the ODS participation-criticism subscale and the scores of psychological capital total, and only one subscale of psychological capital, namely, self-efficiency (Geçkil et al., 2016; Geçkil and Koçyigit, 2017). The ODS transparency subscale correlated positively and weakly with the psychological capital total score and with nearly all of its subscales (Geçkil et al., 2016; Geçkil and Koçyigit, 2017). In contrast to Geçkil and Koçyigit (2017) and Geçkil et al. (2016) identified no significant relationships between the ODS justice and equality subscales. Significantly positive and weak correlations occurred between the ODS accountability subscale and nearly all indicators of psychological capital (Geçkil et al., 2016; Geçkil and Koçyigit, 2017).

Organizational democracy is expected to play a potential role that affects organizational commitment (Allen and Meyer, 1996). So many things could be affected by the lack of OD including employees' commitment to their organization. According to Harrison and Freeman (2004), organizational democracy can help to foster commitment to the organization since, through OD, employees could develop the ability to influence the organization in which they work. By increasing participation in decision-making decisions can be implemented in a smoother way, as well as the commitment of employees toward the final adoption can be increased. Three studies (8.1%) examined the relationship between ODS and organizational commitment (Naldöken and Limoncu, 2019; Uysal, 2019; Yalçinkaya, 2019). Two studies reported significant and positive associations, and one study exhibited no correlation between the total scores of ODS and organizational commitment. The work of Naldöken and Limoncu (2019), and Yalçinkaya (2019) found no relation between continuance commitment and ODS total and the ODS subscales, though Yalçinkaya (2019) identified affective commitment associated with the ODS equality subscale, and normative commitment negatively related to ODS participation-criticism and positively to ODS transparency. Naldöken and Limoncu (2019) revealed significant positive relationships between ODS total and all ODS subscales and all indicators of organizational commitment except continuance commitment.

Organizational silence can deeply affect important areas of the organization such as organizational change, development, transformation of the organization into a pluralistic structure, and decision making (Morrison and Milliken, 2000). In the event that subordinates give incorrect or insufficient feedback or provide no feedback not at all, the organization cannot perceive its own objective position and, thus, will be negatively affected (Milliken et al., 2003). Organizational democracy can break the silence by enabling the employee to criticize what is going on around him. Two studies examined the relationship between ODS and organizational silence. In these two studies, a negative and significant relationship appeared between the total of ODS and organizational silence scores (Erkasap, 2020; Karadağ and Geçkil, 2020). In one study, negative and significant relationships were found between acquiescent silence and ODS participation-criticism (β = −0.274; *p* = 0.001) transparency (β = −0.310; *p* = 0.001), and justice subscales (β = −0.134; *p* = 0.023) (Erkasap, 2020). In another study, negative and significant (*r* = −0.156 to −0.218) relationships arose between acquiescent silence and ODS participation-criticism, equality, accountability, and transparency subscales (Karadağ and Geçkil, 2020). In this study, positive and significant relationships were found between quiescent (defensive) silence and only the participant-criticism subscale (Karadağ and Geçkil, 2020). Negative correlations were found between defensive silence and equality and justice subscales (Erkasap, 2020). Negative and significant relationships were present between the pro-social (protective) silence and ODS total and its subscales (Karadağ and Geçkil, 2020). On the other hand, Erkasap (2020) found a weak positive correlation between pro-social silence and the accountability subscale.

Organizational identification is a type of psychological attachment that occurs when members take on key characteristics of the organization as defining characteristics for themselves (Dutton et al., 1994, p. 242). It can be expected that employees who work in organizations that create a democratic climate would be able to identify with their organizations. Two studies examined the relationship between ODS and organizational identification (Kesen, 2015a,b). Both studies found significant, positive, and moderate correlations between ODS subscales and organizational identification (*r* = 0.304–0.482).

Additionally, one study each demonstrated positive and significant relationships between ODS indicator scales and social capital (Aykanat and Yildiz, 2018), intrapreneurship tendency (Öge and Çiftçi, 2017), organizational justice, organizational support (Bakan et al., 2017), political sensitivity (Karatepe, 2019), quality of work-life (Geçkil and Şendoğdu, 2021), and employee performance (Kesen, 2015b).

One relevant study also found negative and significant relationships between most ODS subscales and organizational depression (Bakan and Gözükara, 2019), and intention to quit the job (Kara, 2020). A negative relationship was reported between the equality and justice subscales of the ODS and the job stress level whereas a weak positive correlation was found between accountability and job stress (Tokgöz and Önen, 2021). Furthermore, the same study showed no significant association between ODS/participation-criticism, ODS/transparency, and job stress.

ODS total score significantly affects work engagement (β = 0.407; *p* = 0.001). The ODS transparency subscale strongly predicted all subscale indicators of work engagement (β = 0.746 to 0.867). Participation-criticism (β = −0.205 to −0.343), justice (β = −0.215 to −0.402), and accountability (β = −0.073 to −0.075) subscales predict the indicators of work engagement negatively (Yildirim and Deniz, 2020). Although the three subscales of ODS (participation-criticism, justice, and accountability) negatively affected the subscales of work engagement, ODS total seemed to positively affect total work engagement. The source of this positive effect on the work engagement total is the very high positive effect of the transparency subscale of the ODS.

Appendix E and Table 5 show the results of three studies with different scale structures. Since the 5-subscale and 28-item structure of the ODS changed in these studies, it seemed appropriate to present these results in a separate table. One of the three studies (Bilge et al., 2020) examined the relationship between OD and employee demographics. No significant relationship emerged between ODS total and subscale scores and gender and age (*p* > 0.05). Married employees had higher ODS participation-criticism subscale scores than singles did (*p* = 0.020). Secondary-school graduates had high ODS justice subscale scores (*p* = 0.039). There is a significant relationship between ODS equality subscale scores and working time (*p* = 0.044). The second study (Can and Dogan, 2020) examined the relationships between OD and ethical leadership and psychological empowerment. A significant correlation was found between ODS total and ethical leadership (*r* = 0.871). Significant correlations were present between the ODS total and the psychological empowerment total (*r* = 0.580), and its autonomy (*r* = 0.462) and impact subscales (*r* = 0.649). The last study (Senol and Aktaş, 2017) examined the relationship between OD and organizational silence. The regression analysis showed organizational democracy positively affecting organizational silence (β = 0.181, *p* = 0.023).

**Table 5:** The correlations and regression of related factors with ODS total and subscales (studies with different scale structures).

**Related factors**			**ODS subscales**				
		**ODS total**	**Participation-critism**	**Transparency**	**Justice**	**Equality**	**Accountability**
		***r/β/R* ^2^**	**r/β**	**r/β**	**r/β**	**r/β**	**r/β**
Bilge et al. (2020)	There is no correlation or regression values since the relationship with OD is not the variable examined						
Can and Dogan (2020)	**Ethical leadership**	0.871^**^					
Can and Dogan (2020)	**Psychological empowerment**	0.580^**^					
	Autonomy	0.462^**^					
	Impact	0.649^**^					
Senol and Aktaş (2017)	**Organizational** **silence**	β = 0.181 *p =* 0.023					

^*^p <0.01,

** *p < 0.001.*

## Discussion

Aiming to examine the organizational democracy levels and related factors for public and private sector employees in Turkey, this focused systematic review investigated the results of 37 studies, with a total of 10,370 employees. The mean of the total ODS scores was found to be 3.30 ± 0.81 (Table 1) for the employees. Considering that the scoring of the scale used a 5-point Likert scale, the participants' ODS scores seem slightly above the “moderate” level and not at the desired level. A score of 4 or more on a concerning 5-point Likert scale indicates a relatively high influence of employees on organizational decision making which may promote their positive organizational attitudes and behaviors (Heller et al., 1998). In the majority of cases, and to varying degrees, OD showed positive correlations with outcomes representing positive organizational behaviors and negative correlations with outcomes assigned to negative organizational behaviors (Appendix D and Table 4). For this reason, taking initiatives to improve OD will benefit both employees and organizations. In this context, examining the factors affecting the establishment of OD in organizations can be a starting point.

As the research outcome shows, the ODS total scores of private-sector employees were higher than those of public-sector employees also in studies that included both (mixed) sectors (see Appendix D). That the OD level of employees in private-sector enterprises in Turkey is higher than those of public-sector employees, represents an expected and significant result that can be explained through the unsuitability of the public sector's bureaucratic structure for establishing OD. The bureaucratic structure was created to meet the needs of a society with a high need for uncertainty avoidance, where high power distance is considered normal (a feature of Turkish social structure mentioned before) (Hofstede, 2021). The institutions of a society with high power distance and high uncertainty avoidance scores must work with detailed rules and a hierarchical structure (Sargut, 2010), as rigid bureaucratic structures. Democratic practices will not easily settle in bureaucratic structures because the rules determine a bureaucratic organizational structure. A social transformation to an organization that can live with uncertainty can weaken bureaucratic structures and enable organic structures to emerge. The transformation of Turkish society into a society that adopts low power distance may also weaken the rigid bureaucratic structures. Ensuring such developments could enable public institutions in Turkey to become more democratic.

ODS participation-criticism subscale scores of the employees (mean = 3.18 ± 0.91) were at a moderate level. The level of participation-criticism of private-sector employees is higher than that of public-sector employees (Table 2). The higher level of participation-criticism of private-sector employees may be due to the enterprises' structural differences. The fact that the score of participation-criticism was at a moderate level can be attributable to the employees of the enterprises in Turkey being unable to participate in decisions sufficiently. They cannot criticize the policies and practices of their institutions, even when finding them incorrect, and the culture of criticism is not sufficiently developed. Insufficient participation of employees in decision-making processes may lead to depriving the organization of their suggestions. In this case, the organization will be managed only by the managers' capacity. That is, the organization will lack the employees' managerial contributions. Increasing the level of employee participation-criticism can lead to positive results for themselves and their organizations. Indeed, three respective studies included in this review (Appendix D and Table 4) found important positive correlations between the levels of employees' participation-criticism and job satisfaction (Kesen, 2015a; Geçkil et al., 2017; Çankaya, 2018), organizational identification (Kesen, 2015a,b), employee performance (Kesen, 2015b), psychological capital (Geçkil et al., 2016; Geçkil and Koçyigit, 2017), perception of organizational support (Bakan et al., 2017), intrapreneurship tendency (Öge and Çiftçi, 2017), social capital (Aykanat and Yildiz, 2018), OCB (Tokay and Eyüpoglu, 2018; Çavuş and Biçer, 2021), political sensitivity (Karatepe, 2019), increase in organizational commitment (Naldöken and Limoncu, 2019), and quality of work-life (Geçkil and Şendoğdu, 2021).

Appendix D and Table 4 further show negative correlations between the ODS participation-criticism level and organizational depression (Bakan and Gözükara, 2019), intention to quit the job (Kara, 2020), the organizational silence total score, and its acquiescent-silence subscale (Erkasap, 2020; Karadağ and Geçkil, 2020). However, mixed findings characterize the association between participation-criticism, defensive silence, and prosocial silence. While in one study no relationship emerged between participation-criticism and job stress (Tokgöz and Önen, 2021), in another study examining the relationships between quality of work-life and ODS (Geçkil and Şendoğdu, 2021), the stress at work subscale (of quality of work-life) and all ODS subscales were positively correlated to a considerable extent. It was expected that ODS would be negatively correlated with stress because organizational democracy includes several resources like job control and autonomy at work supposed to buffer stressing events at the workplace (Ashley et al., 2011). Some of the research results were contrary to this expectation. Organizational context factors (e.g., the economic situation of a company or features of the manufacturing technology) may play a role. Against the background of the unexpected result, new specific research on the unresolved relationship in democratic organizational settings is recommended. Participation-criticism correlation with work engagement was significantly negative (Yildirim and Deniz, 2020). This result, which contradicts the theory, suggests that this relationship should be re-examined through different samples, too.

The ODS transparency subscale scores of the employees (mean = 3.42 ± 0.86) seem slightly above the medium level, and the score of the private-sector employees is higher than the score of the public employees This finding suggests that the employees' scores for transparency regarding their organizations are reasonably good but open to improvement. Strengthening transparency will ensure that the employee is perceived as a shareholder/stakeholder in the decision processes of the organization (Geçkil and Tikici, 2015). Increasing transparency can positively affect the corporate culture, in terms of communication and openness. Thus, the concerned findings indicate positive correlations between the level of transparency and job satisfaction (Kesen, 2015a; Geçkil et al., 2017; Çankaya, 2018), organizational identification (Kesen, 2015a,b), psychological capital (Geçkil et al., 2016; Geçkil and Koçyigit, 2017), perception of organizational support (Bakan et al., 2017), intrapreneurship (Öge and Çiftçi, 2017), social capital (Aykanat and Yildiz, 2018), predominantly with OCB (Tokay and Eyüpoglu, 2018; Barutçu, 2019), political sensitivity (Karatepe, 2019), increase in normative organizational commitment (Naldöken and Limoncu, 2019; Yalçinkaya, 2019), all subscales of work engagement (Yildirim and Deniz, 2020), and quality of work-life (Geçkil and Şendoğdu, 2021). Mixed results leave open the relationship between ODS transparency and affective commitment; no relationship to continuance commitment was identified. Associations with prosocial silence seem unclear and did not exist with defensive silence. Moreover, negative correlations with organizational depression (Bakan and Gözükara, 2019), acquiescent organizational silence (Erkasap, 2020; Karadağ and Geçkil, 2020), and the intention to quit the job (Kara, 2020) were evident. Again, a positive (Geçkil and Şendoğdu, 2021) or no relationship was found between transparency and job stress (Tokgöz and Önen, 2021).

The ODS justice subscales indicated the lowest level among all dimensions (mean = 3.17 ± 0.91). Justice scores of private-sector employees were higher than those of public workers, but the difference was not statically significant (Table 2). Additionally, the respected studies demonstrated positive correlations among the level of ODS justice with job satisfaction (Kesen, 2015a; Geçkil et al., 2017; Çankaya, 2018), organizational identification (Kesen, 2015a,b), perception of organizational support (Bakan et al., 2017), organizational commitment (Naldöken and Limoncu, 2019), employee performance (Kesen, 2015b), social capital (Aykanat and Yildiz, 2018), OCB (Tokay and Eyüpoglu, 2018; Çavuş and Biçer, 2021), political sensitivity (Karatepe, 2019), and quality of work-life (Geçkil and Şendoğdu, 2021). On the other hand, we observed negative correlations between ODS justice and organizational depression (Bakan and Gözükara, 2019), work engagement (Yildirim and Deniz, 2020), intention to quit the job (Kara, 2020). Mixed results leave open the relationship between ODS justice and several forms of psychological capital (Geçkil et al., 2016; Geçkil and Koçyigit, 2017), organizational commitment (Naldöken and Limoncu, 2019; Yalçinkaya, 2019), organizational silence (Erkasap, 2020; Karadağ and Geçkil, 2020), and job stress (Geçkil and Şendoğdu, 2021; Tokgöz and Önen, 2021). Organizational justice is an important determinant of attitudes, decisions, and behaviors (Gilliland and Chan, 2009, p. 167). The fact that the employees have a high level of justice perception regarding the organizations for which they work may produce important positive organizational and behavioral outcomes for both themselves and their organizations. Business managers should determine and implement policies that will increase their employees' perception of justice. The opinions of the employees about the fairness of their treatment affect their organizational commitment (Carmona et al., 2010, p. 210). Studies have found that justice correlates with job satisfaction, evaluation of superiors, trust in management, and turnover intentions (Gilliland and Chan, 2009, p. 172).

ODS equality subscales measurements demonstrated the highest values among all dimensions (mean = 3.47 ± 0.74) from the ODS subscales (Table 1). Equality scores of private-sector employees were higher than from public employees (Table 2). Since equality manifests in the form of rights that laws, statutes, and other general regulators at the institutional level provide, being the highest values to emerge in this dimension seems natural. Equality is equal treatment of those with equal conditions (Geçkil and Tikici, 2015). It is an egalitarian approach to evaluating all employees, regardless of religion, language, race, age, or gender, by considering the value they provide to the business (Bozkurt, 2012). Employees sensing egalitarian policies in their organizations can lead to an increase in positive organizational and behavioral outcomes and a decrease in negatively evaluated outcomes. Studies reveal positive correlations between the ODS equality subscale and job satisfaction (Kesen, 2015a; Geçkil et al., 2017; Çankaya, 2018), organizational identification (Kesen, 2015a,b), political sensitivity (Karatepe, 2019), organizational support (Bakan et al., 2017), social capital (Aykanat and Yildiz, 2018), OCB (Tokay and Eyüpoglu, 2018; Barutçu, 2019), predominantly organizational commitment (Naldöken and Limoncu, 2019), and quality of work-life (Geçkil and Şendoğdu, 2021). However, mixed findings relate equality to psychological capital (Geçkil et al., 2016 versus Geçkil and Koçyigit, 2017) organizational silence (Erkasap, 2020; Karadağ and Geçkil, 2020), intrapreneurship (Öge and Çiftçi, 2017) and job stress (Geçkil and Şendoğdu, 2021; Tokgöz and Önen, 2021). Negative correlations appeared between employees' scores for equality, organizational depression (Bakan and Gözükara, 2019), intention to quit the job (Kara, 2020), and organizational dissent (Erkasap and Ülgen, 2020). The findings let assume that managers developing egalitarian policies and ensuring that employees notice such existing policies can contribute to the formation of several positive outputs for businesses whereas it seems still unclear how equality is related to silence behavior or stress at democratic workplaces.

Employees' level of accountability is slightly above the medium level (mean = 3.27 ± 0.95) (Table 1). The accountability scores of private-sector employees were higher than those of public employees (Table 2). The concerned studies included in this review suggest that as the level of accountability increases, positive organizational attitudes, behaviors, and competencies of employees increase—for example, job satisfaction (Kesen, 2015a; Geçkil et al., 2017; Çankaya, 2018), organizational identification (Kesen, 2015a,b), psychological capital (Geçkil et al., 2016; Geçkil and Koçyigit, 2017), perception of organizational support (Bakan et al., 2017), intrapreneurship (Öge and Çiftçi, 2017), social capital (Aykanat and Yildiz, 2018), predominantly OCB (Geckil and Tikici, 2016; Tokay and Eyüpoglu, 2018; Barutçu, 2019; Çavuş and Biçer, 2021), political sensitivity (Karatepe, 2019), and quality of work-life (Geçkil and Şendoğdu, 2021). Mixed results leave open the relationship between ODS accountability and organizational commitment (Naldöken and Limoncu, 2019; Yalçinkaya, 2019), organizational silence (Erkasap, 2020; Karadağ and Geçkil, 2020) or work engagement (Yildirim and Deniz, 2020). Increase in accountability is associated with a decrease in one negative organizational and behavioral outcome, such as organizational depression (Bakan and Gözükara, 2019) but with an increase in the other negative outcome, namely job stress (Geçkil and Şendoğdu, 2021; Tokgöz and Önen, 2021). Because of the mostly positive effects, developing a culture of accountability and practices in organizations seems recommendable.

In their majority, the five studies that examined the relationship between ODS and OCB revealed moderate or strong correlations between these two variables (Appendix D and 5). OCB, positive extra-role behaviors that employees develop toward the organization, provides various positive contributions at individual, group, and organizational levels (Podsakoff and Mac Kenzie, 1997). OCB reduces the time that managers spend on conflict-management activities by strengthening the social structure of the organization, reducing conflicts and frictions, and maintaining peace (Organ, 1988; Podsakoff et al., 2000), improving performance by increasing organizational effectiveness (Podsakoff et al., 2000). Weak and negative relationships emerged between ODS subscales and the OCB altruism subscale (Geckil and Tikici, 2016). In the same study, the OCB sportsmanship dimension did not show significant relations with ODS and all subscales. In one study, negative relationships arose between the OCB courtesy subscale and ODS subscales (Çavuş and Biçer, 2021). Significant and positive relationships existed with two other OCB subscales (conscientiousness and civic virtue). Conscientiousness includes working hard, obeying rules and regulations, going beyond the minimum role definitions (Podsakoff et al., 2000), and protecting the resources of the organization (Organ, 1988). Civic virtue expresses interest in the organization as a whole. The findings of the reviewed studies confirm that in this dimension, the individual shows the strongest citizenship-oriented behavior between himself/herself and the organization (Organ, 1988; Podsakoff et al., 2000). These dimensions closely relate to the democratic organizational environment that OD creates. In particular, courtesy and civic virtue behaviors closely relate to the culture and socio-moral atmosphere of the organization. Thus, OD can play a key role in increasing OCB, providing important outputs for businesses.

Deep differences arose between the findings of the two studies (Geckil and Tikici, 2016; Çavuş and Biçer, 2021) examining the relationships between ODS and OCB subscales. The second of these studies did not explain the reason for its differences with the first study. The reason for the discrepancy between the findings might have been sample, sector, and method differences. Both studies used similar methods (though the data-collection method is different. In the first study, data were collected through face-to-face interviews with a questionnaire. In the second study, the questionnaire form was sent to the participants). Sample characteristics show similarities. However, sample differences cannot be ignored. In the study by Geckil and Tikici (2016), 60.3% of the sample was female, and in that of Çavuş and Biçer (2021), ~40.9% of the sample was female. Geckil and Tikici (2016) state that the decrease in the level of altruism due to the increase in women's OD level may relate to the nursing profession, which constituted a large part of the female sample, as well as it may relate to gender. They suggested that the fact that the nursing profession is primarily based upon assisting/caregiving to the needy could also explain the altruistic behaviors among nurses (Geçkil and Tikici, 133). However, the finding that emerged in the related study and needed explanation is the inverse correlation between altruistic behavior and ODS and its subscales. The democratic environment may lead to an increased expectation of altruistic behavior toward women, and in this case, women may have responded to this excessive expectation in the opposite direction. The main reason for the disparity between the two studies seemed to be the differences between the sectors. The samples consist of the public health sector (service sector) employees on the one hand (Geckil and Tikici, 2016) and, on the other, private-sector industrial enterprise employees (Çavuş and Biçer, 2021). There are clear organizational-structure differences between the two sectors, including long hours of night shifts for health-sector workers in Turkey and many patients for each staff member. Accordingly, health-sector workers, especially nurses, are asked to make sacrifices in a tiring working environment. The other sample encompasses white-collar workers in private-sector industrial enterprises, consisting of decision-makers and those who work in managerial positions in the institutions for which they work. Presumably, they will not be exposed to external influence for demonstrating altruistic behavior, but they will exhibit this behavior dependent on their inner motivation. Cultural differences, along with organizational-structure differences in both samples, lead to differences in courtesy behavior. In Turkish culture, high power distance is considered normal (Hofstede, 2021), and courtesy can appear as weakness (especially for managers). Since the second sample consisted of white-collar private-sector employees, a negative correlation might emerge between courtesy behaviors and OD.

Participation-criticism is a democratic competency, and its use will lead to an increase in the quality of the organization. In addition to these individual and organizational outputs, the increase in the level of employee participation-criticism can also provide social outputs through political democracy, by improving their most basic democratic competencies. Weber et al. (2020) reveal that individually perceived participation positively affects employees' job satisfaction and prosocial work behaviors. Due to all these positive outputs, we recommend increasing the level of employee participation-criticism of enterprises in Turkey. The low level of participation-criticism among both public- and private-sector employees may relate to the social culture in Turkey. Carrying out studies to fully reveal the factors affecting the level of participation-criticism and determining how to improve it will be useful. Ways of encouraging participation-criticism and making it a part of business culture should be sought. Highlighting and rewarding those who openly express their ideas and suggestions as positive role models can be a method of improving participation and criticism.

In this systematic review, the relationship between ODS and organizational dissent covers five studies. Organizational dissent is any kind of protest and opposition behavior that occurs as a result of dissatisfaction with the practices within the organization, symbolizing a break from the organizational status quo (Kassing and Dicioccio, 2004). Displaced dissent behavior, the third of the three dimensions of organizational dissent, does not provide sufficient validation within the model (Kassing and Dicioccio, 2004). For this reason, studies were examined that concern the two other dimensions (upward dissent and lateral dissent). The absence of organizational dissent leads to the restriction of innovation power (Aytekin, 2019), a decrease in the learning abilities of organizations, a decrease in the diversity of perspectives, and a weakening of relations between the organization and the employee. Organizational identification, organizational citizenship, and job satisfaction are also positively associated with organizational dissent (Kassing et al., 2018).

All three concerned studies found a significantly positive relationship of low to high extent between organizational dissent total and ODS total (Bilyay et al., 2020; Erdal, 2020; Erkasap, 2020). In one of two concerned studies (Bilyay et al., 2020), the ODS total score and the upward dissent score showed a significantly positive and moderate correlation while, in the other study (Erkasap, 2020), Considered separately, ODS participation-criticism does not influence upward dissent, whereas two from threes studies (Erkasap, 2020; Erkasap and Ülgen, 2020; Pelenk, 2020) found a positive, significant, and moderate relationship between the transparency subscale of the ODS and the upward dissent subscale. In two from threes studies studies, a negative, significant, and moderate relationship existed between the ODS justice and accountability subscales and the upward dissent subscale (Erkasap, 2020; Erkasap and Ülgen, 2020 vs. Pelenk, 2020). The fact that employees do not feel the need to give negative feedback to their superiors in businesses where the perception of justice is high, and the culture of accountability is established, can explain this. Upward dissent means that employees share their discontent and stand against their superiors' policies. This type of opposition is desirable opposition for organizations; it is open dissent and contributes to the solution by revealing the problem or the perception of the problem. Arguably, managers can develop upward dissent with OD or with transparency practices, especially. However, not every manager will tolerate opposition and desire its emergence. For the inverse correlation of justice and accountability subscales with upward dissent, justice, and accountability eliminate the need for opposition. This result can imply that if the employee has a strong perception of justice and accountability, he or she does not need to oppose.

Lateral dissent is the opposition to the organizational decisions by members at the same level, which does not directly affect the organizational decision-making and implementation processes. Employees may resort to lateral dissent, fearing the consequences of punishment, rejection, and being ignored or put in a situation where they will feel ashamed if their opinions reach the managers (Zaini et al., 2016). In addition, the employee performs this behavior thinking that he/she is perceived as an enemy or competitor and believing that opposition cannot happen vertically (Özdemir, 2011; Erkasap, 2020). Lateral dissent is not a desirable type of opposition, and the factors leading to it must be reduced. Two of three relevant studies demonstrate that the ODS transparency and equality subscales affect lateral dissent negatively, significantly, and moderately (Erkasap, 2020; Erkasap and Ülgen, 2020; Pelenk, 2020). In other words, perceiving high transparency and equality may decrease lateral dissent among employees. Notwithstanding that, the ODS participation-criticism dimension has a positive, significant, and moderate-to-weak effect on lateral dissent subscale scores (Erkasap, 2020; Erkasap and Ülgen, 2020; Pelenk, 2020). The fact that participation-criticism leads to an increase in lateral dissent contradicts the theoretical knowledge. Participation in management and decisions will likely increase the culture of criticism in the organization and direct the employees toward upward dissent instead of lateral dissent. Contrary to the theoretical structure, this situation requires examination in future studies.

All three studies that examine the relationship between total scores or subscale scores of ODS and job satisfaction identified positive, significant, and moderate-to-strong relationships. These results clearly demonstrate that OD is an important tool for business managers who want to increase job satisfaction. High levels of job satisfaction cause positive effects, such as strong job performance (Spector et al., 2009, p. 39; Judge et al., 2001), organizational commitment, increased organizational citizenship behaviors, decreased absenteeism, and high-level life satisfaction, toward the work, the workplace, and the individual. On the other hand, low-level job satisfaction causes an increase in staff turnover rate, absenteeism, and intention to quit the job (Geçkil et al., 2017, p. 652).

Three studies (Geçkil et al., 2016; Geçkil and Koçyigit, 2017; Karagöz and Atilla, 2018) examined the relationship between total scores of ODS and psychological capital, which reflects the individual's positive psychological state. They found significantly positive correlations of weak-to-moderate size. In sum, the same is true for the relationship of ODS participation-criticism, ODS-transparency, and ODS accountability with different components of psychological capital, whereas the correlations with the ODS-justice and ODS-equality subscales show an unclear picture. Sample differences and structural differences in the organizations where the studies were conducted can explain the different results in these two studies. In the first study (Geçkil et al., 2016), the transparency and accountability dimensions of the ODS related to the individual's psychological capital level, while the justice and equality dimensions were completely unrelated. Participation-criticism shows mixed results. About 90% of the participants in this study worked in public organizations. Employees in the public organization in Turkey continue to work, as long as they do not quit that job voluntarily, and their career paths are foreseeable and, compared to the hospitality industry, public organizations are stable. Certain legal regulations guarantee the rights related to business life (e.g., income, compensation, days off) in public institutions. The sample in this study consisted of physicians and nurses, and 80% of them were high-qualified employees who have undergraduate and graduate degrees. The sample of the second study (Geçkil and Koçyigit, 2017) consisted of employees in hospitality businesses, completely private-sector businesses. It consisted of less-educated and less-qualified employees than the sample of the first study. Regarding the sample of the second study, laws offer less protection for the working conditions and social rights of the employees. The effect of the law protecting the rights of state employees may have superimposed potential effects of the ODS justice and equality dimensions on the psychological-capital levels.

Taking these findings as a whole, psychological capital, with positivity at its center and affected by a positive socio-moral atmosphere, is an important value for organizations in achieving sustainable competitive advantage. This result is a guide for organizations and managers who want to improve their employees' psychological-capital levels.

Studies show that OD positively affects the socio-moral atmosphere, positive organizational behavior patterns of employees, and organizational commitment (Weber et al., 2008, 2009). In three studies examining the relations between the total scores of ODS and organizational commitment, positive correlations were found (Naldöken and Limoncu, 2019; Uysal, 2019; Yalçinkaya, 2019), but the two relevant studies that considered subscales of both concepts demonstrated mixed results. No relationship was found between the continuance commitment subscale and the ODS total score and its subscales. Continuance commitment refers to commitment based on the employee's recognition of the costs associated with leaving the organization (Allen and Meyer, 1996). The priority for employees high on this form of commitment is the extrinsically motivating cost of leaving which seem to be independent from the more intrinsically needs fulfilling possibility to work in a democratic organization. Thus, the findings are compatible with the theory. While positive relationships of weak-to-moderate size were found between affective commitment and ODS total and all ODS subscales in one study (Naldöken and Limoncu, 2019), in another study, affective commitment was only positively affected by the equality subscale (Yalçinkaya, 2019). Naldöken and Limoncu (2019) found positive and significant relationships between normative commitment and ODS and its subscales, while another study (Yalçinkaya, 2019) found negative and significant relationships between normative commitment and ODS participation-criticism. According to theory, OD will likely affect organizational commitment positively. Organizational commitment levels of employees vary according to personal, external, and organizational factors (Northcraft and Neale, 1990). OD directly relates to only one of these three elements (organizational factors), making significant changes in that field. These mixed results are partly inconsistent with theory that assumes that affective and normative commitment are positively influenced by OD (see the meta-analysis by Weber et al., 2020). Examining the relations between organizational commitment and ODS with new studies in the context of Turkish organizations will be useful.

Employee voice is one of the dimensions of OD (Yazdani, 2010; Vopalecky and Durda, 2017; Han and Garg, 2018). Findings of two relevant studies show that as ODS total scores increase, organizational-silence total scores decrease (Erkasap, 2020; Karadağ and Geçkil, 2020). The significant relationships between organizational silence and OD are in line with the literature. Employees making their voices heard is important, in terms of both expressing themselves and conveying their knowledge, skills, and experiences to the organization's management. However, Senol and Aktaş (2017) found that the increase in employees' OD level leads to an increase in the level of organizational silence. According to OD theory, this unexpected result may relate to the small sample size and sample characteristics (94.3% blue collar in the textile industry). Further, regarding specific associations between ODS subscales and different forms of organizational silence, the two relevant studies in the systematic review demonstrate only mixed results. OD revealed the effect of reducing organizational silence in general. The decrease in organizational silence is closely related to the ability of employees to raise their voices. The latter is closely related to other factors such as their personality traits, job stress in the sector, leadership behaviors and workforce qualifications. Thus, examining the effect of OD on the forms of organizational silence requires more studies in that respect. Not all problems with organizational structure may go unnoticed by managers. Accordingly, the voice of the employees will support the managers in noticing the problems. The tendency of the findings is to assume a decrease in the level of silence, in parallel with the increase in democratic practices. This decrease may not be entirely due to OD. However, arguably, employees in a democratic structure will feel more comfortable expressing themselves. It means that employees can express what they think about their environment and report to their superiors what they consider to be wrong. In addition, a more humanist organizational structure requires raising the voice of employees (Pircher-Verdorfer et al., 2012).

Both studies examining the relationship between ODS and organizational identification found moderate correlations (Kesen, 2015a,b). Organizational identification, expressed as employees' feelings of being integrated into their organizations and seeing the success of their organization as their own success, positively affects individual performance (Carmeli et al., 2007). According to Kerr (2004), intra-organizational democratic practices can play an important role in increasing employee performance in service sectors, such as retail, where individualized and one-to-one customer relations are important. Placing OD in organizations makes it more likely that the level of organizational identification of employees, and therefore individual and organizational performance, can increase.

Though based on one study only (Pelenk, 2020), the focused systematic review has revealed relevant results between ODS subscales and types of organizational culture. The participation-criticism, transparency, and justice subscales of ODS show a weak negative correlation with clan culture. According to Cameron and Quinn (1999, p. 36), typical characteristics of clan-culture organizations are teamwork, personnel investment programs, and the organization's commitment to personnel. In a clan-type culture, the community spirit toward a common purpose is very strong, and the organization is like an extended family. According to this theory, participation-criticism, transparency, and justice subscales of ODS will likely show a positive relationship with clan-type culture. However, in a clan-type culture, the organizational system can be a priority over the individual. This approach, which pushes the individual to the second row, can explain the negative relationship. Market culture, another organizational culture types, was positively associated with all subscales of ODS. Since the market culture focuses on suppliers and customers, the positive correlations are theory-conforming (employees may act as collective entrepreneurs). Some positive correlations between the hierarchical type of culture and participation-criticism, equality, and accountability subscales of ODS are surprising. Hierarchy culture represents mechanical and bureaucratic organizations, difficult to describe as compatible with OD theory. However, order and rules are important in hierarchical cultures, and it is clear who will do what and how (Cameron and Quinn, 1999, p. 33). Ensuring participation and accountability with certain rules can help us explain this relationship. On the other hand, equality refers to the regulation and procedures of the rights of the employees, depending on the rules within the organization.

In this systematic review, one study each examined the relationship of further variables with ODS. These studies demonstrate that ODS showed a positive relationship with variables that could have a positive effect on the organization and the employee, such as social capital (Aykanat and Yildiz, 2018), intrapreneurship tendency (Öge and Çiftçi, 2017), organizational justice, organizational support (Bakan et al., 2017), political sensitivity (Karatepe, 2019), quality of work-life (Geçkil and Şendoğdu, 2021), employee performance (Kesen, 2015b). Further, all subscales of ODS were related to organizational depression in one study (Bakan and Gözükara, 2019). While the justice and equality subscales of ODS affected job stress levels negatively, accountability affected them weakly positively (Tokgöz and Önen, 2021). The ODS total score and several ODS subscales and employees' intention to quit the job also showed negative correlations (Kara, 2020). Those variables that showed negative correlations with the total score and the subscales of the ODS could negatively impact employees and the organization. Because each of these variables was examined in only one study in Turkish organizations, the level of evidence for these emerging relationships is low; for this reason, a gap exists which suggests to explore these relationships and compare the findings with those in other country contexts.

Several studies in this systematic review found a relationship between age and OD which indicates that the higher OD perception of employees aged 40 and over may relate to seniority. The perceived higher OD level of seniors may be traceable to their heightened involvement in decision-making processes. In addition, the level of forgiveness increases with age. Increased tolerance levels may also be associated with higher OD perception. Considering the positive results of the psychological and behavioral variables discussed above for employees and organizations, it may be desirable that all employees experience a high-level perception of OD. Studies involving corresponding interventions (e.g., giving employees more information, including them in decision-making processes, creating an organizational climate in which they can raise their voices) can be conducted to increase the OD perceptions of other age groups.

In 6 of the 7 studies that found a significant relationship between OD and gender. The higher OD level of men may relate to their organizational roles (such as the fact that managers are mostly men). In the working world, women are less likely to be brought to management positions and generally work at the lower levels of the career ladder (Sampson and Moore, 2008; Babic and Hansez, 2021). The lower OD level of women may relate to this. In this review, only one study found that the OD level of female employees was higher than that of male employees. The sample of this study was academicians at state universities. Many regulations regarding working life in universities are guaranteed by law. The rate of female academics in Turkey is 38.2% (O'Neil et al., 2019), relatively high compared to other organizations. Different perceptions of the OD level of women and men in their institutions may also differ according to their individual expectations. These results suggest that women's expectations about OD in their organizations may be higher than men's. Future studies may focus on examining women's expectations about OD and ways to increase OD levels.

### Strengths and Limitations

The strengths of this focused systematic review chiefly rest on the inclusion of a large number of studies (*N* = 37) conducted in Turkey, using the same measurement tool, a large number of participants (*N* = 10,370), and the fact that the studies cover various types of businesses in the private and public sectors. The studies included seem rich in terms of numerous variables examined in relation to ODS. The results of this review provide important data for researchers in the field of organizational participation and behavior research. On the other hand, the existence of so many variables and results limited the discussion of each finding in detail. Until now, very few meta-analyses and systematic reviews on organizational psychological outcomes of OD exist (see Weber et al., 2020). This situation also limited the comparison and discussion of research findings. This study used a systematic-review methodology, focusing on studies conducted in one country and with only one method of data-recording. A further limitation appears in the fact that findings for several outcomes are based on only one or two studies. Although systematic review methodology is employed, the limited scope of the present review study is incompatible with the comprehensive core concept of a systematic review. The articles included are observational or correlational studies with a cross-sectional single-source design. Moreover, some articles do not report scale scores. For example, in one study on OCB correlations were reported, but scale scores were not. Such cases, in which the data are only partially available were not excluded, even though they were of low or moderate quality, to reach as comprehensive a dataset as possible. This can be considered a limitation, but to lessen this limitation, missing data were requested from the authors, but none were obtained (Benlioglu, 2021 at Appendix C).

Three studies report a change in the factor structure of the ODS as a result of the Explanatory Factor Analysis. These studies are analyzed separately from the others in Appendix E and Table 5 (Senol and Aktaş, 2017; Bilge et al., 2020; Can and Dogan, 2020). Supporting the construct validity of the ODS scale, the scale structure changed in only three from 37 studies. This change may be due to the small number of participants or cultural differences in the studies. Apart from these, in two studies (Yildirim and Deniz, 2020; Çavuş and Biçer, 2021) that the present study included, the equality subscale of the ODS scale was removed, but the other subscales were left as they were, and ODS scale was used. In these two studies, the researchers state that the reverse items (21 and 23) in this subscale distorted the equality subscale. One of the reasons for the different scale structures may be that these two items.

## Conclusions

This focused systematic review concludes that the OD perceptions of Turkey's public- and private-sector employees are slightly above the scale mean score. The results of this study show that private-sector employees have a higher democratic level. Despite this, the OD perception of both private and public employees is below the assumed “good” level and should be improved. To varying degrees, positive relationships prevail between OD and several outcomes (e.g., job satisfaction, organizational commitment, OCB, psychological capital) that can positively affect the individual, the organization, and the public—that is, OD may strengthen these outcomes. On the other hand, the results suggest negative associations between some phenomena that may cause negative effects on the individual, the organization, and the society (e.g., job stress, organizational depression, and intention to quit) and the perception of OD. The level of OD was higher among men and participants over the age of 40. Overall, these results let us recommend that managers should include democratic practices (e.g., increase participation in decision-making, anchor a culture of criticism, transparency) in their organizations to increase the OD perception of employees.

## Data Availability Statement

The raw data supporting the conclusions of this article will be made available by the authors, without undue reservation.

## Author Contributions

The author confirms being the sole contributor of this work and has approved it for publication.

## Conflict of Interest

The author declares that the research was conducted in the absence of any commercial or financial relationships that could be construed as a potential conflict of interest.

## Publisher's Note

All claims expressed in this article are solely those of the authors and do not necessarily represent those of their affiliated organizations, or those of the publisher, the editors and the reviewers. Any product that may be evaluated in this article, or claim that may be made by its manufacturer, is not guaranteed or endorsed by the publisher.
