# Supplementary material for: Perceived Organizational Democracy and Associated Factors: A Focused Systematic Review Based on Studies in Turkey
Source: Front Psychol. 2022 Apr 15;13:767469. doi: 10.3389/fpsyg.2022.767469 (PMC9051443; doi:10.3389/fpsyg.2022.767469)
Supplement: Supplementary file 1 [file Presentation_1.pdf]

Appendix A

Turkish Form of the Organizational Democracy Scale (Original Scale)

	No	Items	Kesinlikle Katılmıyorum	Katılmıyorum	Kararsızım	Katılıyorum	Kesinlikle Katılıyorum
Katılım-Eleştiri	1.	Örgütsel kararlara katılmam için yöneticiler beni cesaretlendirir					
	2.	Kurumsal karar almada çoğunluğun görüşleri dikkate alınır					
	3.	Kurumumda kararlar alınırken o kararlardan etkilenecek herkesin söz hakkı vardır					
	4.	Yöneticiler hoşlarına gitmese de çoğunluğun verdiği kararlara saygı gösterirler					
	5.	Yanlış bulduğum karar ve politikaları rahatlıkla eleştirebilirim					
	6.	Yönetim, çalışanları eleştiri yapma konusunda cesaretlendirir					
	7.	Yönetim çalışanların eleştirilerini dikkate alır					
	8.	Çalışanların yönetimin uygulamalarını eleştirmesi normal karşılanır					
Şeffaflık	9.	Toplantılarda herkese düşüncelerini ifade etme fırsatı verilir					
	10.	Kurumumda işler şeffaflık ilkesiyle yürütülür					
	11.	Yöneticiler önemli gelişmelerin yaşandığı dönemlerde bilgilendirme toplantıları düzenlerler					
	12.	Kurumumda açık ve çift yönlü bir iletişim vardır					
	13.	Kurumumda performans değerlendirmeleri şeffaflık ilkesine uygun yapılır					
	14.	Kurumum çalışanların eğitim alarak gelişmesini destekler					
Adalet	15.	Kurumumda adil bir ödül sistemi vardır					
	16.	Çalışanların ücret ve diğer gelirleri yaptıkları işe ve kuruma katkıları dikkate alınarak belirlenir					
	17.	Görev dağılımında liyakat dikkate alınır					
	18.	Kurumumuzda değerlendirme kriterleri standartlaşmıştır					
	19.	Amirlerin başarı düzeyinin belirlenmesinde astların görüşleri etkilidir					
Eşitlik	20.	Kurumumda cinsiyet ayrımı gözetilmez					
	21.	Kurumuma personel alımında kişilerin politik düşünceleri ve dünya görüşü etkili olur					
	22.	Toplantılarda, gelen önerinin kimden geldiğine değil önerinin niteliğine bakılır					
	23.	Kurumumdaki çalışanlar arasında ayrımcılık yapılmaktadır					
	24.	Kurumumuzda dil, din, ırk vb. ayrımı gözetilmez					
	25.	Kurumumun demokratik bir örgüt olduğunu düşünüyorum					
Hesap Verebilirlik	26.	İşyerimizde politika ve prosedürler çalışanlar tarafından her zaman sorgulanabilir					
	27.	Kurumumda her düzeydeki çalışandan her zaman hesap sorulabilir					
	28.	Kurumumda hesap verebilme kültürü gelişmiştir					

Appendix B

English Form of the Organizational Democracy Scale (Translated)

		ORGANİZATIONAL DEMOCRACY SCALE<br>Scale items	Strongly disagree	Disagree	Neither agree or disagree	Agree	Strongly agree
Participation-Criticism	1	Managers encourage me to participate in organizational decisions.					
	2	The opinions of the majority are taken into account in institutional decision making.					
	3	Everyone who will be affected by those decisions has the right to speak when decisions are made in my institution.					
	4	Managers respect the decisions made by the majority, even if they don't like it.					
	5	I can easily criticize decisions and policies that I consider wrong.					
	6	Management encourages employees to voice criticism.					
	7	Management takes criticism by employees into consideration.					
	8	It is considered to be normal to criticize management practices by employees.					
Transparency	9	At meetings, everyone is given the opportunity to express his/her thoughts.					
	10	The works are carried out in regards to the principle of transparency in my organization.					
	11	Managers organize informative meetings during periods of important developments.					
	12	There is an open and two-way communication in my organization.					
	13	Performance assessments are conducted in a transparent way in my organization.					
	14	My organization supports the development of employees through training.					
Justice	15	My organization has a fair reward system.					
	16	The wages and other incomes of the employees are determined by taking into account their contributions in their works and to the organization.					
	17	Merit is taken into account when assigning positions.					
	18	Evaluation criteria are standardized in our institution.					
	19	The opinions of subordinates are effective in determining the success level of supervisors.					
Equality	20	There is no gender discrimination in my organization.					
	21	People's political thoughts and worldview are effective in recruiting personnel to my organization.					
	22	At the meetings, the ideas to improve quality are taken into account regardless of who suggest it.					
	23	There is discrimination among employees in my organization.					
	24	Discrimination based on language, religion or race is not accepted in my organization.					
	25	I think my institution is a democratic organization.					
Accountability	26	Policies and procedures in our workplace can always be questioned by employees.					
	27	Employees at all levels in my organization can always be held accountable.					
	28	A culture of accountability has been developed in my institution.					

Appendix C

Studies Considered but Excluded from the Systematic Review

Excluded because sample overlap

Bakan, İ., and Gözükara, H. (2019). Örgütsel demokrasi ve iş tatmini arasındaki ilişki: Bir alan çalışması. In İksad 3. Uluslararası sosyal bilimler kongresi tam metin kitabı, eds. M. F. B. Alodalı, S, Khadhraoı (Adıyaman: İksad publishing house), 444-459

Bakan, İ., Kara, E., and Güler, B. (2017). Örgütsel demokrasi algısının çalışanların iç girişimcilik performansına etkileri: Marmaris'teki otel işletmelerinde bir alan araştırması. HAK-İŞ Uluslararası Emek ve Toplum Dergisi, 6:14, 115-138

Perçin, N.S., Günden, Y., and Çavuşoğlu, M. (2018). The Demographic variables evaluation of organizational democracy perception in Cappadocia. Çobanoglu et al.: Advances in Business, Hospitality, and Tourism Research: Volume 1, 176-182.

Excluded because lack of data

Benlioğlu, B. (2021). Psikolojik güvenin rol içi performansa etkisinde çalışan sesliliği ve örgütsel demokrasinin rolü. [dissertation]. Ankara: Başkent Üniversitesi.

Structural Change of ODS

Cinel, M. O., and Karaman, G. (2019). Meslek odalari yöneticilerinin kurumsal demokrasi algilari ve insani yardim eğilimleri: ordu ili örneği: IV. Uluslararası Demokrasi Sempozyumu, 05-06 Aralık 2019, Proceeding book 25-45, E-KİTAP, ISBN: 978-605-4239-95-5.

Öztürk, M., and Demirtaş, H. (2019). Örgütsel demokrasi bağlamında sendikal demokrasinin sendikal bağlılığa etkisi: Izmir ilinde bir inceleme. İş ve Hayat. 5(9). 9-35.
