# Supplementary material for: Perceived Organizational Democracy and Associated Factors: A Focused Systematic Review Based on Studies in Turkey
Source: Front Psychol. 2022 Apr 15;13:767469. doi: 10.3389/fpsyg.2022.767469 (PMC9051443; doi:10.3389/fpsyg.2022.767469)
Supplement: Supplementary file 2 [file Presentation_2.pdf]

**Appendix D** Descriptive characteristics and main results of studies included in the systematic review

| **Authors, Publication year** | **Type of Article** Manuscript, Unpublished Thesis, Conference Paper, Working Paper<br><br>**Language** English Turkish | **Sector Type:** Public Private<br><br>**Sector** Education Health Banking Hospitality Textile Other | **N=Sample Size** F: Female M: Male | **Educational Status**<br><br>PS: Primary school, HS: High school, G: Graduate, PG: Postgraduate PhD.: Doctorate | **Chronbach's Alpha Reliability Coefficient** ODS Total (Sub-scale Cronbach's Alpha values were taken for studies that did not give ODS Total Cronbach's Alpha)<br>PC: Participate-Criticism<br>T: Transparency,<br>J: Justice,<br>E: Equality,<br>A: Accountability | **Variables searched to be Related to Organizational Democracy** | **Main Results** |
|---|---|---|---|---|---|---|---|
| 1. Kesen, 2015a. | Paper/<br><br>English | Private/<br><br>Retail Sector | N=142<br>F:62<br>M: 80 | PS: 9<br>HS: 80<br>G: 53 | .954 | -Organizational identification<br>- Job satisfaction | - A significant and positive correlation was found between ODS subscales and Organizational Identification (r=.304-.433).<br>- Significant and positive correlations were found between ODS subscales and Job Satisfaction (r=.237-495). |
| 2. Kesen, 2015b. | Paper/<br><br>Turkish | Private/<br><br>Retail Sector | N=174<br>F:85<br>M: 89 | PS: 11<br>HS: 95<br>G: 68 | .954 | - Employee performance<br>- Organizational identification | - A significant and positive correlation was found between ODS subscales and Employee Performance (r=.272-.362).<br>- A significant and positive correlation was found between ODS subscales and Organizational identification (r=.375-482). |
| 3. Geçkil, İleri, Kaya and Karadağ, 2016. | Paper/<br><br>English | Public and Private/<br><br>Hospital Employees (Physician and Nurse) | N=363<br>F:232<br>M:131 | HS: 73<br>G: 191<br>PG: 99 | .933 | - Psychological capital<br>- Demographics (profession, age, educational status, gender, marital status, experience in hospital) | - A significant and positive correlation was found between ODS total and psychological capital (r=.126).<br>- Significant and positive correlations were found between ODS subscales and psychological capital (r=.015-.174).<br>**Demographics**<br>- A significant relationship was found between ODS total and profession against nurses (t=-2.589; p=.010)<br>- A significant correlation was found between ODS total scores and age (F=-2.912; p=.034). ODS scores of individuals under the age of 40 are lower than those of those over the age of 40.<br>- It was determined that there was no significant relationship between ODS scores and education, marital status, and working time (p>.05). |
| 4. Geckil and Tikici, 2016. | Paper/<br><br>English | Public/<br><br>Hospital Employees (Physician, Nurse, Laborant and officer) | N=582<br>F=351<br>M=231 | HS: 61<br>G: 319<br>PG: 202 | .940 | - Organizational citizenship behaviors (OCB)<br>- Demographics (Gender, age, educational status, profession, experience in hospital,) | - A significant and negative correlation was found between ODS total and subscales and OCB Altruism subscale (r= -.118 to -.193).<br>- A significant and positive correlation was found between ODS total and subscales and OCB Conscientiousness subscale (r=.379-.682).<br>- A significant and positive correlation was found between ODS total and subscales and OCB Courtesy subscale (r= .470-.786).<br>- There was no significant correlation between ODS total and subscales of OCB's Sportsmanship subscale (p>.05).<br>- A significant and positive correlation was found between ODS total and subscales and OCB Civic Virtue subscale (r= .531-.892). |

| | | | | | | | |
|---|---|---|---|---|---|---|---|
| | | | | | | | **Demographics**<br>- A significant correlation was found between ODS total and gender in favor of males (t=-3.920; p=.000)<br>- A significant relationship was found between ODS total and profession against nurses (F=-3.132; p=.025)<br>- A significant correlation was found between total ODS and working time (F=-8.683; p=.000). ODS scores of those working for less than 1 year were found to be significantly higher than the others.<br>- It was determined that there was no significant relationship between the participants' ODS scores and their education and age (p>.05). |
| 5.Bakan, Güler and Kara, 2017. | Paper/<br>Turkish | Private/<br>Hospitality Industry | N=201<br>F:88<br>M: 113 | PS: 5<br>HS: 24<br>G: 172 | PC:.809<br>T: .793<br>J:.750<br>E:.689<br>A:.679 | - Organizational justice<br>- Perceived organizational support | - A significant and positive correlation was found between ODS subscales and organizational justice subscales (r= .478-.769).<br>- A significant and positive correlation was found between ODS subscales and organizational support (r= .365-.714). |
| 6.Geçkil, Akpınar and Taş, 2017. | Paper/<br>Turkish | Public/<br>Hospital Employees (Physician, Nurse,Lab orant and officer) | N= 405<br>F:268<br>M: 137 | Unknown | .944 | - Job satisfaction<br>- Demographics (profession, age, educational status, gender, marital status, experience in hospital, income) | - A significant and positive correlation was found between ODS total scores and job satisfaction total (r= .622).<br>- Significant and positive correlations were found between ODS subscale scores and job satisfaction subscales (r= .391-.559).<br>**Demographics**<br>- A significant correlation was found between ODS total score and gender in favor of males (t=--2.929; p=.004).<br>- A significant relationship was found between ODS total score and profession against nurses (F=-10.235; p=.000)<br>- A significant relationship was found between ODS total score and working time (F= -3.037; p=.029). ODS total scores of those who have worked for less than 5 years are higher than the others.<br>- A significant correlation was found between the total ODS and age (F= -3.106; p=.046). ODS scores of individuals under the age of 40 are lower than those of those over the age of 41. |
| 7.Geçkil and Koçyiğit, 2017. | Paper/<br>Turkish | Private/<br>Hospitality Industry | N=144<br>F: 41<br>M: 102<br>Unknown: 1 | PS: 34<br>HS: 60<br>G: 42<br>PG: 5<br>Unknown: 3 | .950 | - Psychological capital | - A significant and positive correlation was found between ODS total and psychological capital total scores (r= .338).<br>- There was no significant correlation between ODS total and subscales and psychological capital optimism subscale scores (p>.05).<br>- Significant and positive correlations were found between ODS total and subscales and psychological capital resilience subscale scores (r= .389-.561).<br>- Significant and positive correlations were found between ODS total and subscales and psychological capital hope subscale scores (r= .227-.301).<br>- Significant and positive correlations were found between ODS total and subscales and psychological capital self-efficacy subscale scores (r= .196-.270). |
| 8.Işık, 2017. | Paper/<br>Turkish | Public/<br>İŞKUR | N=32<br>F: 14<br>M: 18 | HS: 2<br>G: 18<br>PG: 12 | .866 | - Demographics (age, gender, marital status, educational status, job positions/profession, | - A significant positive correlation was found between the age of the participants and the ODS accountability subscale (r=.506; p=.003) and equality subscale scores (r=.401; p=.023). As age increases, scores increase. |

| | | | | | | | |
|---|---|---|---|---|---|---|---|
| | | (an employment agency) | | | | income, and job tenure). | - A negative significant correlation (r= -.468; p=.007) was found between the education level of the participants and the accountability subscale scores of the ODS.<br>- A positive and significant correlation (r= .607; p=.000) was found between the participants' working time and the accountability subscale scores of the ODS. |
| 9.Işıkgöz, Esentaş and Dinçer, 2017. | Paper/<br><br>English | Public/<br><br>Education Sector | N= 191<br>F: 72<br>M:119 | G: 161<br>PG: 30 | .910 | - Demographics (gender, marital status, educational status, school type) | **Demographics**<br>- There was no significant relationship between ODS subscales scores and gender, school type, marital status, educational status (p>.05).<br>- Significant relationships were found between ODS transparency, justice, and accountability subscales scores, and assigned geographical region (p<.05).<br>- A significant correlation was found between ODS equality subscale scores and duration of the experience (Chi-square: 11.319(df:5); p=.045). The equality subscale score of employees between 1-5 years is higher than those with 21-25 years of experience. |
| 10.Öge and Çiftçi, 2017. | Paper/<br><br>Turkish | Private/<br><br>Automotive Textile | N= 77<br>F:52<br>M:24<br>Unknown: 1 | PS: 36<br>HS: 30<br>G: 8<br>PG: 1<br>Unknown:2 | No Information | - Intrapreneurship tendency | - A significant and positive correlation was found between ODS total and intrapreneurship tendency total scores (r= .668).<br>- Significant and positive correlations were found between ODS total and subscales and intrapreneurship tendency innovation subscale scores (r= .267-.521). No significant relationship was found with the ODS justice subscale.<br>- Significant and positive correlations were found between ODS total and subscales and intrapreneurship tendency risk-taking and proactivity subscale scores (r= .268-.663). ODS equality subscale did not show a significant correlation.<br>- Significant and positive correlations were found between ODS total and subscales and intrapreneurship tendency autonomy subscale scores (r= .291-.471). ODS did not show any significant correlation with equity and justice subscales. |
| 11. Atalay, 2018. | Paper/<br><br>English | Public/<br><br>The Provincial Directorates of Youth Services and Sports | N=920<br>F:322<br>M: 598 | PS: 16<br>HS: 112<br>G: 757<br>PG: 24<br>PhD.: 11 | .787 | - Demographics (age, gender, marital status, educational status, profession and geographical region). | **Demographics**<br>- A significant relationship was found between ODS total, ODS justice, and accountability subscales scores and the education levels of the participants (p<.05). Participants with Ph.D. education levels had significantly higher ODS total, justice, and accountability subscale scores (p<.05).<br>- A significant relationship was found between the ODS transparency subscale and occupation. Coaches' transparency subscale scores were found to be significantly higher (p<.05).<br>- Some significant relationships were found between ODS total and subscale scores according to geographical region (p<.05). |
| 12. Aykanat and Yıldız, 2018. | Conference Paper/<br><br>Turkish | Public/<br><br>Municipality | N=120<br>F: Unknown<br>M: Unknown | Unknown | PC:.810<br>T: .880<br>J:.810<br>E:.660<br>A:.850 | - Social capital | - Significant and positive correlations were found between ODS subscales and social capital structural (r= .556-.645), relational (r= .303-.591) and cognitive (r= .460-.688) subscale scores. |
| 13.Çankaya, 2018. | Paper/ | Public/ | N=200<br>F:76 | HS: 84<br>G: 84 | PC:.875 | - Job satisfaction | - Significant and positive correlations were found between ODS subscales job satisfaction total scores (r= .449-.563). |

| | | | | | | | |
|---|---|---|---|---|---|---|---|
| | Turkish | Hospital Employees (Physician, Nurse, Laborant and officer) | M:124 | PG: 32 | T: .924<br>J:.878<br>E:.754<br>A:.794 | - Demographics (profession, age, educational status, gender, marital status, experience in hospital, income) | - Significant and positive correlations were found between ODS subscales and job satisfaction intrinsic satisfaction subscale scores (r= .411-.498).<br>- Significant and positive correlations were found between ODS subscales and job satisfaction extrinsic satisfaction subscale scores (r= .457-.602).<br>**Demographics**<br>- A significant correlation was found between the participants' ODS total scores and gender in favor of males (t=-3.332; p=.001).<br>- A significant correlation was found between the participants' ODS total scores and their marital status in favor of singles (t=-3.837; p=.000).<br>- A significant correlation was found between ODS total and age (F=-10.259; p=.000). ODS scores of individuals under the age of 30 are lower than those of those aged 31-40.<br>- A significant relationship was found between the total ODS and educational status (F=-10.259; p=.000). The ODS score of those whose education level is High School is lower than vocational school.<br>- A significant relationship was found between the total ODS and experience in the hospital (F=-5.377; p=.000). Participants with less than 5 years of experience have a lower ODS total score than those with 11-15 years of experience. |
| 14. Karagöz and Atilla, 2018. | Paper/<br><br>Turkish | Public and Private/<br><br>Banking Sector | N=142<br>F:72<br>M: 70 | G: 131<br>PG: 11 | . 943 | - Psychological capital | - A significant positive correlation was found between ODS total and psychological capital total scores (r=.391; p=000). |
| 15. Tokay and Eyüpoglu, 2018. | Paper/<br><br>English | Private/<br><br>Banking Sector | N=240<br>F:116<br>M:124 | HS: 25<br>G: 128<br>PG: 87 | .790 | - Organizational citizenship behaviors (OCB)<br>- Demographics (Gender, age, educational status, maritial status, experience in banking sector) | - Significant and positive correlations were found between ODS total and subscales and organizational citizenship behaviors total scores (r= .201-.404).<br>**Demographics**<br>- It was determined that there was no significant relationship between the participants' ODS scores and gender, age, education in status, marital status, and experience in the banking sector (p>.05). |
| 16. Bakan and Gözükara, 2019. | Conference Paper/<br><br>Turkish | Public/<br><br>Municipality | N=181<br>F: 42<br>M: 138 | HS: 41<br>G: 114<br>PG: 14<br>Unknown: 11 | PC:.914<br>T: .900<br>J:.866<br>E:.788<br>A:.835 | - Organizational depression | - Significant and negative correlations were found between ODS subscales and organizational depression scores (r= -.601 to -.714). |
| 17. Barutçu, 2019. | Master Thesis/<br><br>Turkish | Private/<br><br>Banking Sector | N=120<br>F: 58<br>M: 62 | G: 84<br>PG: 36 | PC:.805<br>T: .718<br>J:.712<br>E:.725<br>A:.786 | - Organizational citizenship behaviors (OCB)<br>- Demographics (age, gender, marital status, | - ODS subscale scores explain 74.1% of the change in OCB (Adjusted $R^2$=.741; p=.000).<br>- ODS transparency (β=.074; p:.030), justice (β=.454; p:.000), equality (β=.501; p:.000), and accountability (β=.105; p:.008) |

| | | | | | | | |
|---|---|---|---|---|---|---|---|
| | | | | | | educational status, profession/position, and experience in job). | subscale scores significantly and positively affect OCB scores (p<.05).<br>- There was no significant correlation between ODS participation-criticism subscale and OCB scores (p>.05).<br>**Demographics**<br>- There was no significant relationship between ODS scores and participants' gender, age, education level, marital status, job position, and duration of the experience (p>.05). |
| 18. Günden, 2019. | PhD Thesis/<br><br>Turkish | Private/<br><br>Hospitality Industry | N=367<br>F: 194<br>M: 173 | HS: 44<br>G: 318<br>PG: 5 | . 865 | - Organizational citizenship behaviors (OCB) | - As a result of the path diagram between ODS and OCB, a significant positive relationship was found. In standardized path coefficients, it was observed that a one-unit increase in ODS resulted in a .40-unit increase in OCB (r=.40; p<.05). |
| 19. Karatepe, 2019. | PhD Thesis/<br><br>Turkish | Public and Private/<br><br>Hospital Nurses | N=300<br>F: 207<br>M: 93 | HS: 113<br>G: 180<br>PG: 7 | .930 | - Political sensitivity<br>- Demographics (age, gender, marital status, educational status, hospital type public or private, income and experience in job). | - Significant positive correlations were found between ODS total and subscales and political sensitivity total (r=.172-.266), knowledge (r=.187-.317), cognition (r=.130-199), participation (r=.119-.202) and, subscales of interest (r=.136-195).<br>- No significant correlation was found between the ODS Justice subscale and political sensitivity cognition subscale scores (p>0.05).<br>**Demographics**<br>- Men's ODS justice subscale scores were found to be significantly higher than women's scores (t=-1.93 p=.04).<br>- Singles had significantly higher ODS total (t=3.70; p=.00) and all subscale scores (p<.05).<br>- ODS equality subscale scores of vocational school graduates were found to be significantly higher than graduate graduates (F=4.10; p=00).<br>- It was determined that the ODS total (t=8.12; p=.00) and all subscale scores of the participants working in private hospitals were significantly higher than the scores of those working in public hospitals (p<.05).<br>- It was found that as the working time of the participants increased, their justice scores decreased significantly (r=-132; p<.05).<br>- A significant negative correlation was found between the income level of the participants and the ODS total (r=-.358; p<.01) and all subscale scores (p<.05). |
| 20. Naldöken and Limoncu, (2019). | Paper/<br><br>Turkish | Public/<br><br>Hospital Employees (Physician, Nurse, Laborant and officer) | N= 326<br>F:170<br>M:156 | PS: 16<br>HS: 75<br>G: 194<br>PG: 41 | .929 | - Organizational commitment<br>- Demographics (gender, age, marital status, educational status, profession and job tenure) | - Positive and significant correlations were found between ODS total and subscales and organizational commitment total scores (r=.302-.446).<br>- Positive and significant correlations were found between ODS total and subscales and organizational commitment affective commitment subscale scores (r=.270-.368).<br>- Positive and significant correlations were found between ODS total and organizational commitment continuance commitment subscale (r=.016). There is no significant relationship between ODS and its subscales and organizational commitment continuance commitment subscale (p>.05).<br>- Positive and significant correlations were found between ODS total and subscales and organizational commitment normative commitment subscale (r=.352-.489). |

| | | | | | | | |
|---|---|---|---|---|---|---|---|
| | | | | | | | Demographics<br>- It was found that there was no significant relationship between ODS scores, gender, and duration of the experience (p>.05).<br>- Singles had significantly higher ODS total and subscale scores (p<.05). |
| 21. Uysal, 2019. | Master Thesis/<br><br>Turkish | Public/<br><br>Sea Transportation Sector | N=316<br>F: 18<br>M: 298 | PS: 54<br>HS: 147<br>G: 114<br>PG: 1 | .922 | - Organizational commitment | - A significant relationship was found between ODS total and organizational commitment total scores (p<.05). |
| 22. Yalçınkaya, 2019. | Master Thesis/<br><br>Turkish | Private/<br><br>Communication Sector | N=397<br>F: 254<br>M: 143 | HS: 140<br>G: 222<br>PG: 35 | PC:.821<br>T: .741<br>J:.782<br>E:.711<br>A:.724 | - Organizational commitment<br>- Demographics (age, gender, marital status, educational status, and experience in job). | - A positive and significant relationship was found between the ODS equality subscale and the organizational commitment affective commitment subscale (β=.225; p:.015).<br>- There was no significant relationship between ODS subscales and continuance commitment subscale (p>.05).<br>- A negative and significant relationship was found between ODS participation-criticism and organizational commitment normative commitment subscale (β= -.376; p:.000).<br>- A positive and significant relationship was found between ODS transparency and organizational commitment normative commitment subscale (β=-.482; p:.000).<br>**Demographics**<br>- Men's ODS justice subscale scores were found to be significantly higher than women's scores (t=2.361 p=.000).<br>- It was determined that there was no significant relationship between ODS scores and participants' age, education level, marital status, and duration of the experience (p>.05). |
| 23. Bilyay, Yalçın and Özpolat, 2020. | Paper/<br><br>Turkish | Public/<br><br>Education Sector | N=202<br>F:135<br>M: 67 | G and PG: 202 | .700 | - Organizational dissent<br>- Demographics (gender, type of school and total employment duration in profession) | - A significant and positive correlation was found between ODS total and organizational dissent total (r=.220), upward dissent subscale (r=.48) scores.<br>- No significant correlation was found between ODS and lateral dissent and displaced dissent subscales (p>.05).<br>- It was found that the upward dissent subscale explained 14.8% of the variance in the ODS (Adjusted R2=.148; p=.001).<br>**Demographics**<br>- It was found that there was no significant relationship between OD and participants' gender and working time (p>.05). |
| 24. Erdal, 2020. | Master Thesis/<br><br>Turkish | Public/<br><br>Educational Sector Teacher | N=345<br>F: 238<br>M: 107 | G: 345 | .767 | - Organizational dissent<br>- Demographics (age, gender, marital status, educational status, and experience in job). | - A positive and significant correlation was found between ODS and organizational dissent (r=.647).<br>**Demographics**<br>- It was determined that there was no significant relationship between ODS and gender, marital status, age, and professional seniority. |
| 25. Erkasap, 2020. | PhD Thesis/<br><br>Turkish | Private/<br><br>Various Sector | N=509<br>F: 352<br>M: 157 | HS: 29<br>G: 202<br>PG: 235<br>PhD: 43 | .939 | - Personality characteristics<br>- Organizational silence<br>- Organizational dissent | - One unit increase in ODS total leads to a .304 increase in organizational dissent total (β= .304; p=.003).<br>- ODS transparency subscale has a positive effect on upward dissent subscale (β= .464; p=.001). On the other hand, justice (β= -.451; |

| | | | | | | | |
|---|---|---|---|---|---|---|---|
| | | | | | | | p=.001) and accountability (β= - .304; p=.001) subscales have a negative impact on organizational dissent.<br>- ODS transparency (β= - .286; p=.001) and equality (β= - .205; p=.001) subscales negatively affect lateral dissent subscale. On the other hand, the participation-criticism subscale positively affects lateral dissent (β= .336; p=.001).<br>- One unit increase in ODS leads to a .309 unit decrease in organizational silence (β= -.309; p=.004).<br>- The participation-criticism (β= -.274; p=.001) transparency (β= -.310; p=.001) and justice (β= -.134; p=.023) subscales of the ODS were acquiescent silence subscales negatively affects the scale.<br>- ODS equality (β= -.162; p=.004) and justice (β= -.305; p=.001) subscales negatively affect the defensive silence subscale.<br>- ODS accountability subscale positively affects prosocial silence subscale (β= .151; p=.001).<br>- Personality characteristic adaptive peace maker (β= .288; p=.005), strict perfectionist (β= .323; p=.001), quiet specialist (β= .189; p=.037), and loyal sceptic subscales (β= .280; p=.007) affect ODS significantly and positively. |
| 26. Erkasap and Ülgen, 2020. | Working Paper/<br><br>Turkish | Private/<br><br>Various Sector | N= 225<br>F: 161<br>M: 64 | HS: 13<br>G: 89<br>PG: 123 | PC:.938<br>T: .917<br>J:.889<br>E:.916<br>A:.815 | - Organizational dissent | - ODS transparency subscale (β= .286; p=.001) on total organizational dissent was positive; justice (β= -.251; p=.001), equality (β= -.150; p=.020) and accountability (β= -.228; p=.001) subscales had a negative and significant effect has been determined. ODS participation-criticism subscale did not affect the total organizational dissent (p<.05).<br>- On vertical organizational dissent, the ODS transparency subscale (β= .459; p=.001) was in the positive direction; justice (β= -.445; p=.001) and accountability subscales (β= -.331; p=.001) were found to have a negative and significant effect. No significant correlation was found between ODS participation-criticism and equality subscales and upward organizational dissent (p>.05).<br>- On organizational dissent, ODS transparency (β= -.192; p=.001) and equality (β= -.262; p=.001) subscales were found to be negative; the participation-criticism subscale (β= .267; p=.001) was found to have a positive and significant effect. It was determined that ODS justice and accountability subscales did not have a significant effect on organizational lateral dissent (p>.05). |
| 27. Kara, 2020. | Master Thesis/<br><br>Turkish | Private/<br><br>Industrial Enterprise | N=300<br>F: 54<br>M: 246 | PS and HS: 192<br>G: 89<br>PG: 19 | .940 | - Intention to quit from job<br>- Demographics (age, gender, marital status, educational status, income, and experience in job). | - A negative and significant correlation was found between ODS total (r=-.418), participation-criticism (r=-.389), transparency (r=-.417), justice (r=-.476), and equality (r=-.165) subscales and intention to quit the job. There was no significant relationship between ODS Accountability subscale and the intention to quit from the job.<br>**Demographics**<br>- Men's ODS participation-criticism (t=-5.548; p=.000) and accountability (t=-4.027; p=.001) subscale scores were found to be significantly higher than women's scores.<br>- As the age of the participants increases, the ODS participation-criticism (F=33.509; p=.000), transparency (F=40.522; p=.000), and justice (F=30.638; p=.000) subscale scores increase significantly. |

| | | | | | | | |
|---|---|---|---|---|---|---|---|
| | | | | | | | - Participants between the ages of 30-39 had significantly higher ODS scores of equality (F=24.033; p=.000) and accountability (F=12.202; p=.000) subscales.<br>- Participants with high school or lower education levels had higher ODS participation-criticism (t=6,000; p=.003) and transparency (t=5.435; p=.016) subscale scores compared to the scores of participants with higher education levels.<br>- Married participants had significantly higher ODS scores on all subscales (t=-.999 to -6.277; p<.05).<br>- ODS participation-criticism (F=69.439; p=.000), transparency (F=122.601; p=.000), justice (F=97.823; p=.000), and equality of participants with work experience of 11 years or more (F=14.406; p=.000) subscale scores were significantly higher than the scores of those with shorter experience. |
| 28. Karadağ and Geçkil, 2020. | Paper/<br><br>Turkish | Public/<br><br>Higher Education Sector | N=192<br>F:87<br>M: 102<br>Unknown: 3 | PG: 192 | .960 | - Organizational silence<br>- Demographics (gender and title) | - Negative and significant correlations were found between ODS total and subscales and organizational silence total scores (r=-.159 to -.258).<br>- Negative and significant correlations were found between ODS total and subscales and acquiescent silence subscale scores (r=-.156 to -.218). There is no relationship between the ODS justice subscale and acquiescent silence.<br>- Negative and significant correlations were found between ODS total and subscales and defensive silence subscale scores (r=-.184 to -.293).<br>- A positive and significant correlation was found between the ODS participation criticism subscale and the prosocial (protective) silence subscale (r=.149). There is no significant relationship between ODS total and other subscales and protective silence subscale.<br>**Demographics**<br>- Women's ODS total (t=2.288; p=.023), transparency (t=2.365; p=.019), justice (t=2.696; p=.008) and accountability subscales (t=3.082; p=.002) scores were found to be significantly higher than that of men. No significant correlation was found between ODS participation-criticism and equality subscale and gender (p>.05). |
| 29. Pelenk, 2020. | Paper/<br><br>Turkish | Private/<br><br>Constructio n Sector | N=380<br>F: 95<br>M: 285 | Unknown | .910 | - Organizational culture<br>- Organizational dissent | - Negative and significant correlations were found between ODS justice and transparency subscales and clan culture subscale (r=-.105 to -.120).<br>- Positive and significant correlations were found between the ODS participation criticism subscale and the adhocracy culture subscale (r=.103).<br>- Positive and significant correlations were found between ODS subscales and market culture subscale (r=.166-.283).<br>- Positive and significant correlations were found between ODS participation, criticism, equality, and accountability subscales and hierarchy subscale (r=.175-.275).<br>- There was no significant relationship between ODS subscales and upward dissent subscale (p>.05). |

| | | | | | | | |
|---|---|---|---|---|---|---|---|
| | | | | | | | - Positive and significant correlations were found between the ODS participation criticism, transparency, and justice subscales and the lateral dissent subscale (r=.216-.263).<br>- Positive and significant correlations were found between the ODS participation criticism, transparency, and justice subscales and the displaced dissent subscale (r=.229-.281). |
| 30.Üst Can, 2020. | PhD Thesis/<br><br>Turkish | Public and Private/<br>Higher Educational Sector | N=281<br>F: 103<br>M: 174 | G: 13<br>PG: 59<br>PhD: 205 | .957 | - Demographics (age, gender, marital status, educational status, university type and experience in job). | **Demographics**<br>- It was determined that there was no significant relationship between ODS and gender, marital status, and professional seniority.<br>- Negative significant correlations were found between ODS accountability subscale scores and age (r=-.137), and education (r=-.121) level (p<.05). |
| 31. Yıldırım and Deniz, 2020. | Paper/<br><br>Turkish | Private | N= 252<br>F: Unknown<br>M. Unknown | PS and HS: 29<br>G and PG: 223 | .943 | - Work engagement | - A one-unit increase in ODS total score causes an increase of 0.407 in work engagement (β= .407).<br>- The increase in ODS participation-criticism subscale scores leads to a significant decrease in work engagement vigor, dedication, and absorption subscale scores (β= -.205 to -.343).<br>- The increase in ODS transparency subscale scores leads to a significant increase in work engagement perseverance, dedication and adoption subscale scores (β= .746-.867).<br>- The increase in ODS justice subscale scores leads to a significant decrease in work engagement perseverance, dedication and adoption subscale scores (β= -.215 to -.402).<br>- The increase in ODS accountability subscale scores leads to a significant decrease in work engagement perseverance and dedication subscale scores (β= -.073 to -.075). |
| 32.Çavuş and Biçer, 2021. | Paper/<br><br>English | Private/<br><br>Various Sector | N=257<br>F: 105<br>M: 152 | HS: 70<br>G: 120<br>PG: 57 | .845 | - Organizational citizenship behaviors (OCB) | - Positive and significant correlations were found between ODS subscales and OCB altruism subscale (r=.357-.431).<br>- Positive and significant correlations were found between ODS subscales and OCB conscientiousness subscale (r=.435-.496).<br>- There were positive and significant correlations between ODS subscales and OCB courtesy subscale (r=.414-.487).<br>- Positive and significant correlations were found between ODS subscales and OCB civic virtue subscale (r=.614-.676).<br>- Negative and significant correlations were found between ODS subscales and OCB sportsmanship subscale (r=-.390 to -.468). |
| 33. Geçkil and Şendoğdu, 2021. | Paper/<br><br>English | Public and Private/<br><br>Banking Sector | N=397<br>F: 171<br>M: 226 | HS: 15<br>G: 323<br>PG: 59 | .960 | - Quality of work life<br>- Demographics (age, gender, marital status, educational status, bank type, job positions/profession, income, and working time in business). | - Positive and significant correlations were found between ODS total and subscales and quality of work-life total (r=.545-.801).<br>- ODS total and subscales, job and carrier satisfaction (r=.451-.676), general well-being (r=.509-.716), control at work (r=.410-.697), working conditions (r=.527-.745), stress at work (r=.359-.521), and home-work interface (r=.459-.735) subscales were found to have positive and significant correlations.<br>**Demographics**<br>- It was found that there was no significant relationship between ODS and gender and educational status (p>.05).<br>- A significant correlation was found between ODS and marital status in favor of singles (t=-2.30; p=.022). |

| | | | | | | | |
|---|---|---|---|---|---|---|---|
| | | | | | | | - A significant correlation was found between ODS and age groups (F=5.400; p=.000). The ODS score of the under-31 age group is higher than those aged 32-37.<br>- The ODS scores of those with a working period of 5 years or less were found to be significantly higher than those with a 6-11 year working period (F=4.893; p=.002). |
| 34. Tokgöz and Önen, 2021. | Paper/<br><br>English | Public/<br><br>Educational Sector | N=622<br>F: 350<br>M: 272 | Unknown | .940 | - Job stress level | - Negative and significant correlations were found between ODS subscales and job stress total (r=-.110 to -.230). There was no significant relationship between the ODS accountability subscale and job stress (p>.05). |
