# Supplementary material for: Perceived Organizational Democracy and Associated Factors: A Focused Systematic Review Based on Studies in Turkey
Source: Front Psychol. 2022 Apr 15;13:767469. doi: 10.3389/fpsyg.2022.767469 (PMC9051443; doi:10.3389/fpsyg.2022.767469)
Supplement: Supplementary file 3 [file Presentation_3.pdf]

Appendix E Descriptive characteristics and main results of studies included in the systematic review (studies with different scale structures).

**Authors, Publication year**	**Type of Article** Manuscript, Unpublished Thesis, Conference Paper, Working Paper<br><br>**Language** English Turkish	**Sector Type:** Public Private<br><br>**Sector** Education Health Banking Hospitality Textile Other	**N=Sample Size** F: Female M: Male	**Educational Status**<br><br>PS: Primary school, HS: High school, G: Graduate, PG: Postgraduate PhD.: Doctorate	**Chronbach's Alpha Reliability Coefficient** ODS Total (Sub-scale Cronbach's Alpha values were taken for studies that did not give ODS Total Cronbach's Alpha) PC: Participate-Criticism T: Transparency, J: Justice, E: Equality, A: Accountability	**Mean and Standart Deviation**	**Variables searched to be Related to Organizational Democracy**	**Main Results**
1. Bilge, H., Barbuta-Misu, N., Zungun, D., and Virlanuta, F.O. (2020).	Paper/<br><br>English	Private/<br><br>Enterprises	N= 209<br>F:73<br>M:136	PS: 16<br>HS: 62<br>G: 135<br>PG: 22	PC: .642<br>T: .795<br>J: .790<br>E: .689	Not reported	-Demographics (gender, age, marital status, educational status, total employment duration in profession, etc.)	- Demographics<br>- There was no significant relationship between ODS total and subscale scores and gender and age (p>.05).<br>- Married employees have higher ODS Participation-Criticism subscale scores than singles (p= .020).<br>- Secondary school graduates had high ODS justice subscale scores (p=.039).<br>- It was determined that there is a significant relationship between ODS equality subscale scores and working time (p=.044).
2. Can, N., and Doğan, İ.F. (2020).	Paper/<br><br>Turkish	Public/<br><br>Higher Education Sector	N=129<br>F:36<br>M: 93	G: 11<br>PG: 118	,984	3.42±1.04	-Ethical leadership<br>-Psychological empowerment	- A positive and significant correlation was found between ODS total and ethical leadership (r=.871).<br>- Positive and significant correlations were found between ODS total and psychological empowerment total (r=.580), Significant correlations were found between the ODS total and its autonomy (r=.462) and impact subscales (r=.649). (r=.462), and impact subscales (r=.649).
3. Şenol, S., and Aktaş, H. (2017).	Paper/<br><br>Turkish	Private/<br><br>Textile Sector	N=130<br>F:107<br>M: 22	HS: 63<br>G: 13<br>PG: 41	PC and T:.836<br>J and E:.551<br>E and A:.537<br>A:.707	2.95±.89	-Organizational silence<br>-Demographics (age, educational status, job tenure,)	- A significant and positive correlation was found between ODS participation-criticism, transparency, equality, and justice subscales and organizational silence relational silence subscale scores (r= .278-321).<br>- A significant and positive correlation was found between ODS equality and accountability subscales and organizational silence defensive silence subscale scores (r= .198).<br>- Demographics<br>- A significant correlation was found between ODS total scores and age (F=2.870; p=.017). Participants over the age of 45 have higher ODS scores.<br>- There was no significant relationship between ODS total scores and education and working time (p>.05).
